# Selenoprotein M Inhibits the Replication of Influenza A Virus by Regulating Reactive Oxygen Species Levels

**DOI:** 10.3390/life15050714

**Published:** 2025-04-28

**Authors:** Minxuan Liu, Jinhui Wang, Weigang Li, Bo Zhao, Yuanyuan Zhang, Qiaoying Zeng, Guangyuan Liu

**Affiliations:** 1 College of Veterinary Medicine, Gansu Agricultural University, Lanzhou 730070, China; mxliu1991@163.com (M.L.); wangjinhui0606@163.com (J.W.); 2 State Key Laboratory of Veterinary Etiological Biology, Key Laboratory of Veterinary Parasitology of Gansu Province, Lanzhou Veterinary Research Institute, Chinese Academy of Agricultural Science, Lanzhou 730046, China; 3 Animal Husbandry and Veterinary Station of Zhenyuan County, Zhenyuan 744500, China; 18719718116@163.com (W.L.); 18294092571@163.com (Y.Z.); 4 Gansu Agriculture Technology College, Duanjiatan 425, Lanzhou 730030, China; zbtaobaozy@gmail.com

**Keywords:** influenza A virus, selenoprotein M, reactive oxygen species, selenocysteine site, inhibited replication

## Abstract

**Background:** Influenza A virus (IAV) is the major pathogen responsible for influenza pandemics and can cause seasonal influenza outbreaks. In general, viral infection of host cells increases reactive oxygen species (ROS) levels, a process that triggers cell death, lung injury (LI), and other damage mechanisms. **Methods:** In our previous study, we revealed that selenoproteins may inhibit IAV replication at the cellular level. In this study, we determined the effect of selenoprotein M (SelM) on Nanoluc-IAV-PR8 replication through Nanoluc analysis. The mechanism through which selenoprotein inhibits the replication of the influenza virus was investigated using the SelM knockout cell line, nano-luciferase reporter assays, RNAi, qPCR, Western blot, and confocal microscopy. **Results:** Our experimental results show that SelM can effectively inhibit the replication of influenza A viruses and could potentially be used as a broad-spectrum inhibitor for IAV therapy in future clinical treatments. The increase in ROS levels induced by IAV infection was found to be inhibited by SelM, which possesses an important Sec functional site, thus weakening the ability of IAV to replicate in cells. **Conclusions:** The results of this study highlight SelM as a selenoprotein that can effectively inhibit IAV replication.

## 1. Introduction

Influenza A virus (IAV), consisting of eight segments encoding at least 17 structural and nonstructural proteins, is a single-stranded, negative-sense RNA virus [1]. IAVs have a wide range of hosts and are the main pathogens responsible for human influenza pandemics due to their ability to mutate and evolve into multiple subtypes, in addition to their high pathogenicity [2,3]. At present, the annual death toll from respiratory diseases caused by IAV stands at approximately 29 to 600,000 [4]. Infected individuals primarily comprise elderly people, children and pregnant women with low immunity, in addition to individuals with chronic diseases of the lung or cardiovascular system. Symptoms of infection include high fever, cough, multi-organ pain (including headache, joint pain, and sore throat) and a runny nose, which can cause general or severe discomfort, and in some cases, death [2,5]. Swine influenza virus and avian influenza virus have the same evolutionary origin as human IAV and belong to the IAV family. Respiratory diseases or systemic symptoms often occur following infection, and in some cases, the mortality rate can reach 100%. These viruses generally infect humans directly across interspecies barriers, causing significant public health issues [6]. Historical influenza pandemics or gene donors are derived from avian influenza virus or new strains recombined with avian influenza virus. The results of a number of previous studies indicate that reactive oxygen species (ROS) play a crucial role in lung injury caused by IAV infection by inducing the apoptosis of infected lung epithelial cells [7,8,9,10].

Selenium, one of the essential trace elements in the human body, is involved in the formation of various enzymes and is the main component of the antioxidant glutathione peroxidase within the body [11]. The common feature of most selenoproteins is that the sequence contains selenocysteine (Sec) residues; these residues are located at the active sites of the enzymes and are used to catalyse redox reactions [12]. The physiological function of selenoproteins is therefore highly dependent on the presence of Sec, and if the Sec is mutated to any other amino acid residue, the enzyme will become inactivated. Research has shown that humans possess 25 selenoproteins [11]. Selenoprotein M (SelM) is a folded endoplasmicreticulum protein similar to thioredoxin that can regulate the amount of selenium in the diet to prevent cancer and regulate the redox balance of the endoplasmic reticulum [13]. Owing to the high expression of SelM in the brain, a number of researchers have investigated the role of SelM in neuroprotection. SelM overexpression in neurons has been shown to prevent the oxidative damage induced by H_2_O_2_; in comparison, the knockout of SelM with siRNA has been shown to induce a decrease in cell viability and strongly induce apoptosis [14].

Selenoproteins play a key role in human health. Evidence from previous studies confirms that selenium deficiency can increase the risk of various diseases, such as cancer [15], cardiovascular diseases [16], diabetes [17], and liver disease [18]. Research on selenium and viruses primarily focuses on its preventive and auxiliary therapeutic effects. One research group used a selenium-containing metal complex drug delivery system, Ru(biim)(PhenSe)2 (RuSe), to investigate its antiviral efficacy against influenza viruses and its potential antiviral mechanisms. Their results showed that RuSe regulates selenium metabolism and increases the expression levels of selenoproteins GPx1 and TrxR1, thereby inhibiting viral replication and assembly [19]. Glycine nano-selenium can enhance the efficacy of avian influenza vaccines [20]. A number of researchers have also investigated the therapeutic effects of selenium nanochelates and their ability to aid in vaccine immunity. Among them, polyethylene glycol (PEG)-modified gray selenium nanoparticles (PEG-SeNPs) were found to inhibit H1N1-induced cell apoptosis through ROS-mediated signaling pathways [21]. Oseltamivir (OTV) surface-modified nanoparticles (SeNPs) (Se@OTV) prevented the binding of H1N1 to host cells by inhibiting hemagglutinin and neuraminidase activity, thus preventing H1N1 infection of MDCK cells and halting chromatin condensation and DNA fragmentation to exert antiviral functions [22]. In another study, surface modification of selenium nanoparticles (NPs) by amantadine (AM) (Se@AM) significantly inhibits H1N1 infection of host cells by inhibiting neuraminidase activity, ROS production, and AKT phosphorylation activation [23].

To date, researchers have yet to investigate the effect of SelM on IAV replication. The results of our previous study revealed that selenium impacts influenza virus replication. The aim of this study was to investigate whether SelM can inhibit IAV replication and its related molecular mechanisms. Although the function of the SelM protein has been partially elucidated, further research is needed to clarify the mechanism through which this protein exerts its functions and impacts IAV replication.

## 2. Materials and Methods

### 2.1. Biosafety and Ethical Statements

Influenza virus research was conducted in a biosafety level 2 laboratory approved for such activities by the Lanzhou Veterinary Research Institute, Chinese Academy of Agricultural Sciences and in strict compliance with the Biosafety Law of the People’s Republic of China [24].

### 2.2. Cells, Viruses, and Plasmids

A549 cells were cultured in Kaighn’s modified Ham’s F-12 nutrient mixture (Gibco, New York, NY, USA) supplemented with 10% FBS and penicillin/streptomycin. HEK293 and U2OS cells were cultured in DMEM (Gibco, New York, NY, USA) supplemented with 10% (volume/volume) FBS (Gibco BRL, 10099-141, New York, NY, USA) and 1 × penicillin/streptomycin (Gibco BRL, 10378016, New York, NY, USA). All cells were cultured and passaged in a 37 °C, 5% CO_2_ incubator. The IAV virus (A/Puerto Rico/8/1934 (PR8, H1N1)) was inoculated and propagated using SPF chicken embryos(Xinxing Dahuanong Poultry and Egg Co., Ltd., Yunfu, China) that had been developing for a period of 10 days. The reconstructed virus in the SPF chicken embryos is stored at −70 °C for future use [1,25]. Nanoluc-IAV-PR8 is a reporter virus constructed and preserved in our laboratory. Reverse genetics technology is primarily used to insert the nano luciferase gene into the 3′ end of the NS1 fragment in the NS gene of H1N1 influenza virus A/PuertoRico/8/34 (PR8) to produce a luciferase reporter virus. Its biological characteristics were evaluated by purifying and passing the recovered recombinant luciferase reporter virus, measuring EID_50_, TCID_50_, and in vivo and in vitro proliferation ability, and using a mouse pathogenicity test. Simultaneously, a recombinant luciferase reporter virus and a small animal in vivo imaging system were used to perform visual analysis of virus proliferation in vivo to assess whether the Nanoluc-IAV-PR8 luciferase gene reporter virus was successfully constructed. See the References for the specific methods employed [26]. The genomic sequence of human Selenoprotein M (SelM/SEMP, Gene ID: 140606), encoding 435 amino acid residues, was acquired from the NCBI, followed by primer design as summarized in Table 1. The sequence was cloned into the pRK expression vector using the prokaryotic expression methods, and the Flag tag was fused into the framework to obtain the Flag-SelM plasmid. An empty vector (EV) was also cloned into the pRK expression vector. The HEK293T cell line was primarily utilized for prokaryotic expression validation and related experimental procedures.

### 2.3. Reagents and Antibodies

The following antibodies were used in this study: HRP-conjugated anti-Flag (A8592) (Sigma, Sigma-Aldrich (Shanghai) Trading Co., Ltd., Shanghai, China), anti-GAPDH (ab181602) (Abcam, Cambridge, UK), Alexa Fluor 488-conjugated anti-mouse IgG (A0428) and Cy3-labelled goat anti-rabbit IgG (A0516) (Beyotime, Shanghai, China). The following reagents were used in this study: NP-40 (ST366), DAPI (C1002), and Passive Lysis 5× Buffer (E1941) (Promega, Madison, WI, USA). In addition, a scrambled negative control RNA (NC) and SelM-specific short interfering RNA (RiboBio Co., Guangzhou, China), Lipofectamine 2000, RNAi MAX, and TRIzol (Invitrogen, Carlsbad, CA, USA), and SYBR Green I Master Mix (Roche, Basel, Switzerland), were also used [24].

### 2.4. si-RNA

Based on the SelM gene, the si-RNA of SelM (si-SelM) was designed, and the target sequence was as follows: GCATCCCACTCAGTGAAAT (detailed in the Supplementary Material “si-RNA sequences report”, line 20, highlighted). The synthesis of the target sequence was performed by Guangzhou RiboBio Co., Ltd. (Guangzhou, China). The si-SelM and a negative control (NC) were transfected into cells, and after 24 h, the cells were washed three times with serum-free medium and infected with IAV or Nanoluc-IAV-PR8, which were detected at different time points according to the experimental requirements.

### 2.5. Nano-Luciferase Reporter Assays

First, 5 × 10^4^ cells/well were seeded into a 24-well plate and cultured overnight to reach 70–80% confluence. They were subsequently infected with the Nanoluc-IAV-PR8 virus. After 24 h, the cells were lysed using 1× Passive Lysis Buffer (Promega, Madison, WI, USA) for 15 min at room temperature. The lysates were transferred to a microplate, and NanoLuc activity was measured by adding Nano-Glo^®^ Luciferase Assay Substrate (Promega, Madison, WI, USA). Luminescence was immediately quantified using a microplate reader.

### 2.6. RNA Isolation and Quantitative PCR

Total RNA was isolated from the cells using TRIzol reagent following the manufacturer’s guidelines. cDNA synthesis from mRNA was performed with M-MLV reverse transcriptase (Promega, Madison, WI, USA), as per the supplied protocol, using GAPDH as a stable internal reference. Gene expression analysis was conducted on an ABI 7500 detection system (Applied Biosystems, Foster City, CA, USA), with relative RNA levels quantified using the 2^−ΔΔCT^ method. The corresponding primer sequences for qPCR analysis are listed in Table 2 [27].

### 2.7. Western Blot Analysis

Protein extraction was performed using RIPA buffer (Beyotime, Shanghai, China). The extracted proteins were separated using 10% SDS-PAGE gels and subsequently transferred onto nitrocellulose membranes (Bio-Rad, Hercules, CA, USA). The membranes were blocked with 5% skim milk in TBST for 1 h at room temperature. Primary antibodies were then applied and incubated overnight at 4 °C. After washing, the membranes were treated with HRP-conjugated secondary antibodies for 1 h. Lastly, protein bands were detected using an enhanced chemiluminescence (ECL) system (GE Healthcare, Boston, MA, USA) [1].

### 2.8. Confocal Microscopy

The expression of SelM in cells was localized using confocal microscopy. Briefly, cells were plated on coverslips in 12-well plates and transfected for 24 h before being harvested. The cells were fixed with 4% paraformaldehyde for 20 min at room temperature, followed by three washes with PBS. Permeabilization was performed using 0.1% Triton X-100 in PBS for 10 min and blocked with 5% skim milk for 1 h. The cells were then incubated with the specified primary and secondary antibodies and DAPI. Imaging was conducted using a Leica confocal microscope (TCS SP8) equipped with a 100× oil immersion objective (NA 1.40) [28].

### 2.9. Intracellular ROS Detection

A549 cells were cultured in 96-well plates until they reached 60–70% confluency, transfected with target plasmids or infected with the virus and incubated for 24 h. For in situ probe loading, DCFH-DA was diluted 1:1000 in serum-free medium (final concentration: 10 μM), added to replace the culture medium (at a volume sufficient to cover the cells; e.g., ≥1 mL/well for 6-well plates), and incubated at 37 °C for 20 min. The cells were washed three times with serum-free medium to remove extracellular DCFH-DA. The ROS-positive control (Rosup, 1:1000 ratio, e.g., 1 μL/1 mL medium) was applied for 20–30 min to induce significant ROS elevation. Fluorescence intensity was quantified using a microplate reader (excitation/emission: 488/525 nm) in real-time or at intervals pre- and post-stimulation.

### 2.10. Statistical Analysis

Data are presented as means ± standard deviations (SDs). Statistical analyses were performed using GraphPad Prism software (version 6.0, GraphPad Software, La Jolla, CA, USA), employing Student’s two-tailed nonparametric *t*-test or analysis of variance (ANOVA). Differences between groups were considered significant at a *p* value of <0.05 (*), <0.01 (**), <0.001 (***), and <0.0001 (****), ns: non-significant.

## 3. Results

### 3.1. SelM Suppresses IAV Replication

**Figure 1:**
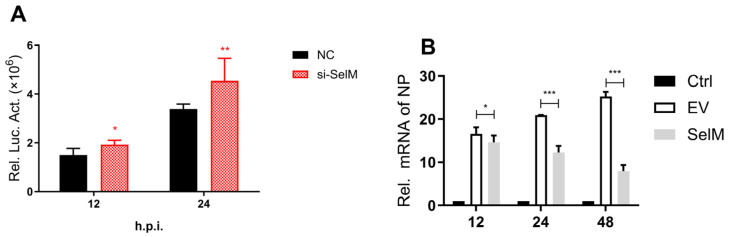

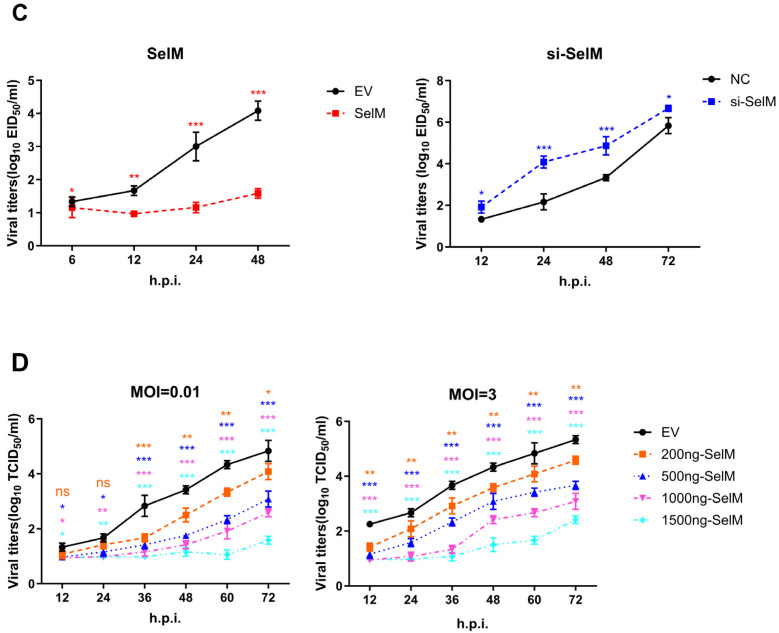
SelM inhibits IAV replication. (**A**) si-SelM or the NC was transfected into the A549 cells, and the cells were then infected with the PR8 virus at a multiplicity of infection (MOI) of 1. (**B**) The SelM expression plasmid or the EV were transfected into A549 cells, respectively. (**C**) The SelM expression plasmid, EV/si-SelM expression plasmid or NC were transfected into A549 cells, respectively. (**D**) Different doses of the SelM expression plasmid or EV were transfected into A549 cells. Abbreviations: NC, negative control; EV, empty vector; Ctrl, control; *, *p* value of <0.05; **, *p* value of <0.01; ***, *p* value of <0.001; ns, non-significant.

In this study, our experimental results showed that SelM effectively inhibited IAV replication. We used nano-luciferase to show that the virus was successfully constructed in the laboratory and that SelM could effectively inhibit IAV replication at 12 h and 24 h after infection (Figure 1A). This phenomenon was confirmed experimentally. To verify that the inhibition of IAV replication was due to SelM, we overexpressed the SelM plasmid and the corresponding siRNA and then infected it with IAV. The mRNA levels of IAV-NP significantly decreased in the experimental group with overexpression induced by the SelM plasmid (Figure 1B); the viral titer was also determined (Figure 1C). Moreover, we used a SelM transfection dose gradient (200 ng, 500 ng, 1000 ng, and 1500 ng), infected the samples with IAV, and detected the viral titer 24 h after transfection (Figure 1D). The results of the two groups revealed that SelM inhibited IAV replication in a dose-dependent manner.

### 3.2. Effect of SelM Knockout on IAV Replication

**Figure 2:**
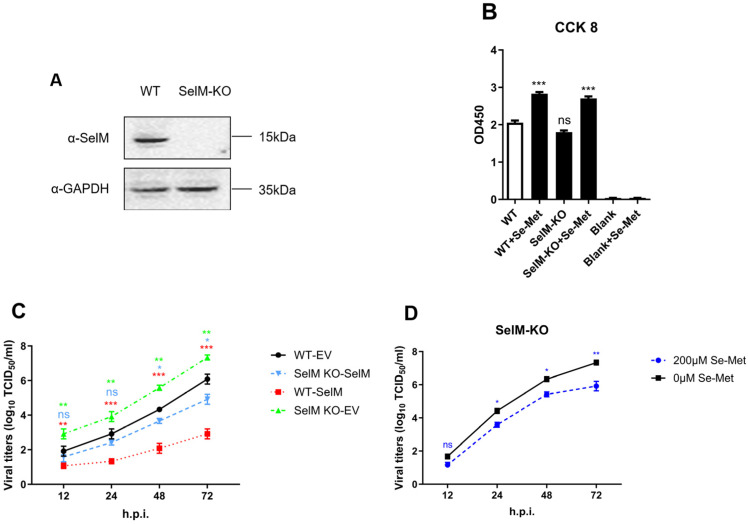
The impact of SelM knockout on IAV replication. (**A**) SelM+/+ or SelM−/−A549 cells and the expression level of endogenous SelM. (**B**) SelM+/+ or SelM−/−A549 cells treated with Se-Met for 24 h were detected with Cell Counting Kit 8 (CCK-8). (**C**) SelM−/−A549 cells were treated with 200 μM of Se-Met or 0 μM of Se-Met. (**D**) SelM+/+ or SelM−/−A549 cells were transfected with the SelM expression plasmid or EV. The viral titer curve between the SelM+/+-EV and SelM+/+-SelM groups is shown in red, that between the SelM+/+-EV and SelM−/−-SelM groups is shown in blue, and that between the SelM+/+-EV and SelM−/−-EV groups is shown in green. Abbreviations: WT, wild-type; EV, empty vector; Ctrl, control; *, *p* value of <0.05; **, *p* value of <0.01; ***, *p* value of <0.001; ns: non-significant.

To further confirm that the inhibitory effect on the influenza virus was due to SelM, we successfully constructed a SelM knockout cell line in our experiment (Figure 2A). In order to ensure that subsequent experiments were not affected, and to verify that the cell activity after SelM knockout (SelM-KO) was not significantly different from that of wild-type A549 (WT-A549) cells and had no effect on the function of Se-Met after entering the cells, we respectively tested the corresponding cell vitality and established controls. Our results confirmed that the cells did not lose vitality due to the knockout of the SelM gene (Figure 2B). Detection of virus replication status after overexpressing the SelM plasmid in SelM knockout cells showed that the knockout cells’ resistance to the influenza virus was significantly reduced compared to wild-type A549 cells. However, after the knockout cells were replaced and transfected with the SelM plasmid, similar results were observed as with the wild-type A549 cells, and the replication level of IAV remained basically unchanged (Figure 2C). The results showed that IAV had stronger replicability in SelM knockout cells, and the effect on IAV replication after overexpressing the corresponding plasmid returned to normal levels. Concurrently, in order to prove the effect of knockout cell lines on IAV replication, we observed a significant difference in the effect of the cell lines on IAV replication after treatment with or without selenomethionine. Selenomethionine treatment was hypothesized to significantly inhibit influenza virus replication; however, it had a much weaker inhibitory effect on IAV (Figure 2D). Our results showed that IAV replication in SelM knockout cell lines was significantly higher than in the control cells, with IAV titers in the experimental group being 1.5–2 times higher than those in the control group. When the corresponding plasmid was overexpressed in the SelM knockout cell lines, the viral titer of IAV returned to a similar level to that before SelM knockout.

### 3.3. Effect of IAV Infection on the Expression and Distribution of SelM

**Figure 3:**
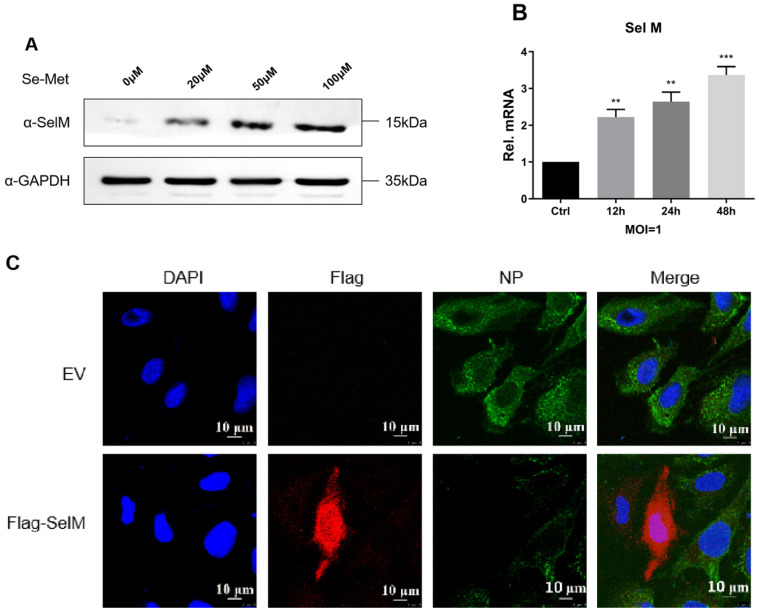
Effects of IAV infection on the expression and distribution of SelM. (**A**) Following pretreatment of A549 cells with different concentrations of Se-Met for 24 h, immunoblotting analysis was performed with SelM endogenous antibodies. (**B**) After A549 cells were infected with the H1N1 virus (MOI = 1), SelM mRNA levels were quantified using qPCR at different times post-infection. (**C**) U2OS cells were transfected with the Flag-SelM plasmid for 24 h and were subsequently left uninfected or infected with IAV for 12 h before being stained with MitoTracker. In the images, red indicates Flag-SelM, and green indicates IAV-NP. Scale bar: 10 μm. Abbreviations: NC, negative control; EV, empty vector; Ctrl, control; **, *p* value of <0.01; ***, *p* value of <0.001; ns, non-significant.

To determine the effects of IAV infection on the protein expression of SelM and its intracellular distribution, we examined SelM protein expression in A549 cells and found that its expression was dependent on selenomethionine concentration (Figure 3A). The mRNA levels of SelM were determined after infection with IAV. The results showed that the mRNA level increased gradually at 24 h after infection (Figure 3B). Laser confocal microscopy analysis revealed that the SelM protein was expressed in both the cytoplasm and nucleus, whereas the IAV-NP protein was not expressed; in comparison, the expression level of the IAV-NP protein did not change in the control group that overexpressed Flag-EV (Figure 3C). Our results demonstrate that IAV replication is significantly inhibited when SelM is highly expressed.

### 3.4. SelM Inhibits IAV Replication by Regulating ROS Levels

**Figure 4:**
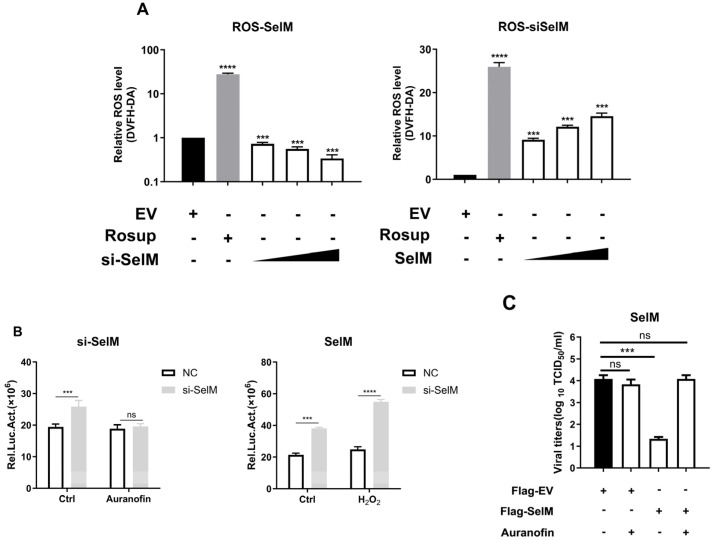
SelM inhibits IAV replication by regulating ROS levels. (**A**) A549 cells were transfected with the EV or increasing amounts of the SelM expression plasmid. (**B**) After transfecting the A549 cells with si-SelM or NC, they were infected with the PR8 virus at an MOI of 1 for 24 h before being treated with auranofin or H_2_O_2_. (**C**) After transfecting the A549 cells with SelM or EV for 24 h, the A549 cells were infected with the PR8 virus under the condition of MOI 1 before being treated with auranofin. Abbreviations: NC, negative control; EV, empty vector; ***, *p* value of <0.001; ****, *p* value of <0.0001; ns, non-significant.

The results of previous studies have confirmed that SelM can regulate the level of ROS. In order to verify the hypothesis that the inhibitory effect of SelM on IAV is caused by ROS, we examined whether SelM affects ROS levels after IAV infection. When SelM siRNA was overexpressed and dose-escalated, ROS levels increased in a dose-dependent manner; in comparison, after overexpressing Flag-SelM, ROS levels decreased with the increase in SelM overexpression, indicating that SelM inhibited ROS levels mediated by IAV infection (Figure 4A). In order to verify this phenomenon, we used the ROS inhibitor auranofin and the stimulant H_2_O_2_ to alter ROS levels. When si-SelM was overexpressed and infected with the Nanoluc-IAV-PR8 fluorescent reporter virus, no significant difference was found in the nanofluorescence values in the auranofin experimental group; in comparison, the nanofluorescence values in the H_2_O_2_ group significantly increased, proving that SelM is indeed able to inhibit ROS levels (Figure 4B). However, when SelM was overexpressed and the ROS inhibitor auranofin was added after IAV infection, viral titration results showed a more than two-fold decrease in viral titer in the SelM group compared to the auranofin treatment group and the control group, suggesting that SelM reduces IAV replication by inhibiting ROS (Figure 4C).

### 3.5. The Sec Site Is Critical for SelM’s Impact on IAV Replication

**Figure 5:**
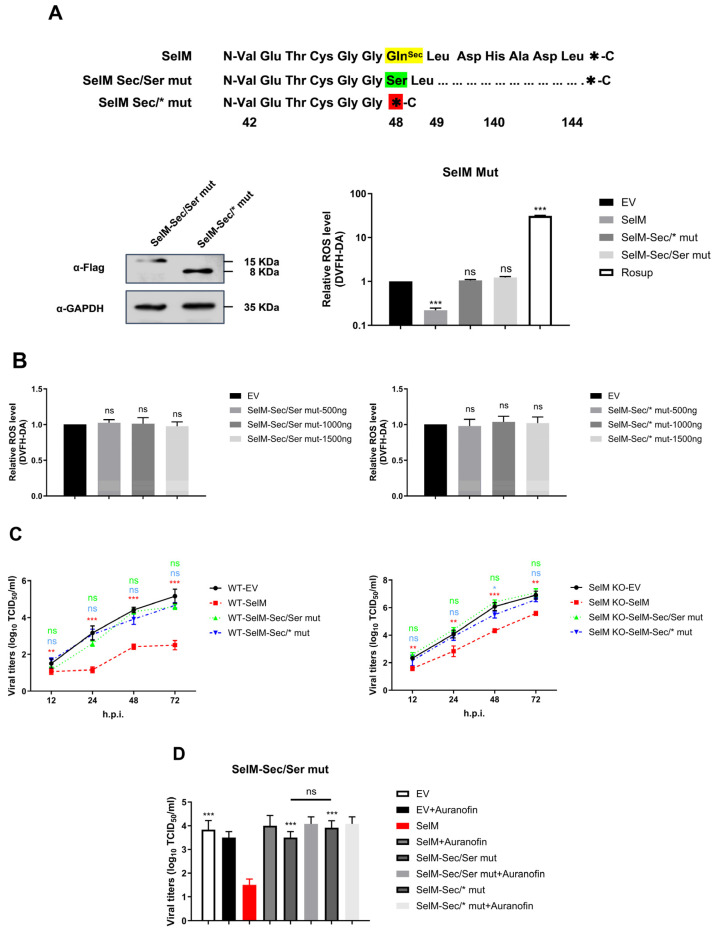
The Sec site is the key to the effect of SelM on IAV replication. (**A**) Design and construction of mutants of the SelM protein (SelM Sec/Ser mut or SelM Sec/*mut) based on the characteristics of Sec and selenoproteins. (**B**) A549 cells were transfected with either the different transfection doses of the SelM Sec/Ser mut or SelM Sec/*mut expression plasmid or EV. (**C**) SelM+/+ or SelM−/− A549 cells were transfected with the SelM, SelM Sec/Ser mut, SelM Sec/*mut expression plasmids, or EV. (**D**) A549 cells were transfected with SelM, SelM Sec/Ser mut, SelM Sec/*mut, or EV and subsequently infected with the PR8 virus at an MOI of 1 for 24 h before the cells were treated with auranofin. Abbreviations: WT, wild-type; EV, empty vector; *, *p* value of <0.05; **, *p* value of <0.01; ***, *p* value of <0.001; ns, non-significant.

SelM reduces the replication of IAV by inhibiting ROS; however, the mechanism through which this protein exerts its function has yet to be elucidated. To confirm that the function of SelM mainly depends on the Sec site, we designed two experimental groups of mutants of the corresponding protein: one in which Sec was mutated to serine, and another in which the Sec-specific stop codon TGA was mutated to the common termination codon TAA. The effects of the mutants on ROS levels were subsequently examined, and the results revealed that the SelM mutants were no longer able to reduce ROS levels (Figure 5A). To determine whether the ROS level was affected by the two groups of mutant plasmids, ROS were transfected with different doses of mutant plasmids. The results showed that the SelM mutant plasmids were not able to alter the ROS level at different doses (Figure 5B). Subsequently, wild-type SelM plasmids and two corresponding mutant plasmids were overexpressed in wild-type A549 cells and SelM knockout cells. After IAV infection, changes in the virus were detected at 12 h, 24 h, 48 h, and 72 h. The results showed that the SelM mutants lost the ability to inhibit IAV replication to some extent in all of the strains compared with the wild-type (Figure 5C). The viral titer of the mutant cells infected with IAV was determined using the ROS inhibitor auranofin at 48 h. Compared with the mutant plasmid group without auranofin, the mutant plasmid group treated with auranofin had no effect on the replication of IAV. The reliance on the Sec site was the main reason for the effect of SelM on the ROS level, and the replication of IAV was inhibited by ROS (Figure 5D).

## 4. Discussion

In this study, we found that the improvement in cell activity was induced by selenomethionine, which prompted our investigation into whether the replication of IAV in cells is inhibited by selenoproteins. In a previous study, researchers focused on 25 known selenoproteins in mammals and designed and synthesized 25 types of selenoprotein siRNAs. After verifying this effect, the effect of selenoprotein on Nanoluc-IAV-PR8 replication was determined through Nanoluc analysis, and selenoprotein-SelM was identified, which has an inhibitory effect on Nanoluc-IAV-PR8. The results presented herein fill this research gap and confirm that selenoproteins can effectively inhibit IAV replication (Figure 1). The results of previous studies have shown that IAV replication is influenced by modulation of ROS levels [29], and SelM reduces the deposition of lipid peroxides caused by IAV infection, thus inhibiting IAV replication through changes in ROS levels.

SelM is one of the inducers of selenium in vivo, is highly expressed in the human brain, and may be involved in antioxidation, neuroprotection, and intracellular calcium regulation. These processes are the key factors that prevent the occurrence and development of Alzheimer’s disease (AD) [29,30]. We first confirmed that the overexpression of SelM significantly inhibited the replication of many subtypes of IAV in cells, and the results of interfering with or knocking out the expression of SelM in cells via siRNA and sg-RNA support this finding (Figure 2). The localization of SelM in cells was also monitored, and it was found that the expression of the IAV-NP protein was significantly inhibited when SelM was highly expressed in cells (Figure 3). These results suggest that SelM inhibits the replication of IAV in many different types of cells.

Although the authors of previous studies have investigated the inhibition of IAV replication through the antioxidant pathway by altering the level of ROS [31,32], researchers have yet to examine how SelM affects the level of ROS and inhibits IAV replication. The results of this study not only confirm that SelM can effectively regulate the level of ROS but also demonstrate that SelM inhibits the replication of IAV in cells by inhibiting the increase in ROS levels caused by IAV infection and maintaining the dynamic redox balance within cells (Figure 4). Despite these findings, the molecular mechanism through which SelM regulates ROS remains unclear.

By elucidating the mechanism and regulation of selenoprotein synthesis [33], we determined the characteristics of selenoproteins with Sec sites. After mutating Sec to a similar serine, we used the SelM-Sec/SerMut plasmid to perform related research. Our results showed that the effect of the mutated SelM was not dependent on the concentration of selenomethionine at the protein level, and the mutant no longer regulated the level of ROS; therefore, it could not inhibit the replication of IAV. After the Sec site-specific UGA stop codon was mutated into a common TAA stop codon, the SelM-Sec/*Mut mutant plasmid was reused, and the results were consistent with those described above. Moreover, to confirm the reliability of the results, two mutant plasmids were overexpressed in SelM knockout cell lines to determine their effects on ROS levels and IAV replication. Our results are in line with our expectations. Therefore, our results show that SelM inhibits IAV replication by regulating the level of ROS and that Sec sites in selenoproteins play an important role in this regard. These results confirm that SelM is a highly promising broad-spectrum inhibitor of IAV (Figure 5). The mechanism through which IAV utilizes ROS remains unclear. The authors of recent studies suggest that when the body is infected with the influenza virus, IAV “hijacks” the biological function of the host cell and enhances viral replication, resulting in tissue damage through the imbalance between the redox control of IV and excess ROS production [34,35]. IAVs induce oxidative stress in the cytoplasm through the AhR pathway, particularly stressing the endoplasmic reticulum (ER) and mitochondria, which in turn results in ROS production [36]. If ROS production exceeds antioxidant levels, the ROS balance is disrupted, and IAV-infected cells undergo apoptosis and necrosis, leading to cell death. If ROS production is inhibited by antioxidants, the REDOX balance can be maintained, ensuring the host cell’s resistance to IAV infection and antiviral immunity [37]. Despite the above findings, further research is needed to assess how to effectively apply SelM for the clinical prevention and treatment of IAV.

Statistics indicate that there are severe selenium deficiencies in China, with only one-third of areas meeting the internationally published standard critical value of 0.1 mg/kg, and two-thirds of areas are classified as selenium deficient. Selenium, an essential trace element, cannot be synthesized or stored in the human body and can only be obtained from external sources. Moreover, as China is a developing country, a key question arises: is there any difference in the resistance to IAV among people living in poor areas characterized by selenium deficiency compared to those living in poor areas without selenium deficiency? In this study, we screened the provinces that meet the definition of poverty-stricken areas, and our results indicated differences in selenium availability, with Guizhou Province being identified as an area not affected by selenium deficiency and Shaanxi Province being identified as a selenium deficiency area. Based on the national statistical report on IAV published by the Public Health Science Data Center (https://www.phsciencedata.cn, accessed on 30 March 2023) covering three consecutive years (2009–2011) [38], although these regions are all poor areas, the incidence of influenza virus (1/100,000) differed between them. The average incidence rate in Guizhou Province from 2009 to 2011 was 3.0181; however, the average incidence rate in Shaanxi Province, which is located in a selenium-deficient area, was 6.0594 from 2009 to 2011. This value is significantly higher than that in Guizhou Province. Based on the analysis of the above data, it can be inferred that people living in poor, selenium-deficient areas are more susceptible to IAV. The results obtained in this study demonstrate that, as one of the selenoproteins that plays a major role in selenium acquisition, SelM deficiency also leads to an increase in the incidence of influenza virus, which is consistent with clinical statistics. Our study findings, therefore, also highlight new directions for the regional prevention of influenza virus infection.

## 5. Conclusions

In summary, the results of this study demonstrate that SelM is a selenoprotein that can effectively inhibit IAV replication. After IAV infection, cells are stimulated to produce oxidative stress, resulting in increased ROS levels and damage to cells, thus promoting further replication of the virus. When SelM is highly expressed in cells, this protein can act as a redox agent to effectively regulate ROS levels and inhibit IAV replication, thereby reducing cellular damage caused by IAV. Our results also confirmed that the Sec functional site is a key site for SelM to regulate ROS levels.

## Figures and Tables

**Table 1:** PCR primers.

Gene	Species	Upstream Primer Sequence (3′-5′)	Downstream Primer Sequence (3′-5′)
*Selenoprotein M (SelM/SEMP* *)*	Human	GACGACGATGACAAGGGGTCGACCATGAGCCTCCTGTTGCCT	GCATGCTCGAGCGGCCGCCTACAGGTCAGCGTGGTCC

**Table 2:** qPCR primers.

**Gene**	**Species**	**Upstream Primer Sequence (3′-5′)**	**Downstream Primer Sequence (3′-5′)**
*GAPDH*	Human	AAAATCAAGTGGGGCGATGCT	GGGCAGAGATGATGACCCTTT
*HPRT1*	Human	GCTTGGATTCCTACAAAGAAGCA	ATAGATGGTCAATGCGGCGTC
*NP*	Human	AGGACAGGGTCCCCCTTGCC	CCTCCAGCCCGCTCACTTGC
*SelM*	Human	AACCGCCTAAAGGAGGTGAAG	GATGCGCTCTAGTTCCTCGTA

## Data Availability

All the data generated during the current study are included in the manuscript. The raw data supporting the conclusions of this article will be made available by the authors without undue reservation.

## Supplementary Materials

The following supporting information can be downloaded at https://www.mdpi.com/article/10.3390/life15050714/s1, Figure S1: The original image and original data of Figure 1; Figure S2: The original image and original data of Figure 2; Figure S3: he original image and original data of Figure 3; Figure S4: he original image and original data of Figure 4; Figure S5: The original image and original data of Figure 5; Table S1: si-RNA sequences report.

## References

[1] Xu S., Han L., Wei Y., Zhang B., Wang Q., Liu J., Liu M., Chen Z., Wang Z., Chen H. (2022). MicroRNA-200c-targeted contactin 1 facilitates the replication of influenza A virus by accelerating the degradation of MAVS. PLoS Pathog..

[2] Fiore A.E., Shay D.K., Haber P., Iskander J.K., Uyeki T.M., Mootrey G., Bresee J.S., Cox N.J. (2007). Prevention and control of influenza. Recommendations of the advisory committee on immunization practics (acip), 2007. MMWR Recomm. Rep..

[3] Krammer F. (2019). The human antibody response to influenza A virus infection and vaccination. Nat. Rev. Immunol..

[4] Zeng Y., Xu S., Wei Y., Zhang X., Wang Q., Jia Y., Wang W., Han L., Chen Z., Wang Z. (2021). The PB1 protein of influenza A virus inhibits the innate immune response by targeting MAVS for NBR1-mediated selective autophagic degradation. PLoS Pathog..

[5] Krammer F., Smith G.J.D., Fouchier R.A.M., Peiris M., Kedzierska K., Doherty P.C., Palese P., Shaw M.L., Treanor J., Webster R.G. (2018). Influenza. Nat. Rev. Dis. Primers.

[6] Osorio J.E., Velez I.D., Thomson C., Lopez L., Jimenez A., Haller A.A., Silengo S., Scott J., Boroughs K.L., Stovall J.L. (2014). Safety and immunogenicity of a recombinant live attenuated tetravalent dengue vaccine (DENVax) in flavivirus-naive healthy adults in Colombia: A randomised, placebo-controlled, phase 1 study. Lancet Infect. Dis..

[7] Oda T., Akaike T., Hamamoto T., Suzuki F., Hirano T., Maeda H. (1989). Oxygen radicals in influenza-induced pathogenesis and treatment with pyran polymer-conjugated SOD. Science.

[8] Akaike T., Ando M., Oda T., Doi T., Ijiri S., Araki S., Maeda H. (1990). Dependence on O^2−^ generation by xanthine oxidase of pathogenesis of influenza virus infection in mice. J. Clin. Investig..

[9] Tantcheva L.P., Stoeva E.S., Galabov A.S., Braykova A.A., Savov V.M., Mileva M.M. (2003). Effect of vitamin E and vitamin C combination on experimental influenza virus infection. Methods Find. Exp. Clin. Pharmacol..

[10] Vlahos R., Selemidis S. (2014). NADPH oxidases as novel pharmacologic targets against influenza A virus infection. Mol. Pharmacol..

[11] Labunskyy V.M., Hatfield D.L., Gladyshev V.N. (2014). Selenoproteins: Molecular pathways and physiological roles. Physiol. Rev..

[12] Aachmann F.L., Fomenko D.E., Soragni A., Gladyshev V.N., Dikiy A. (2007). Solution structure of selenoprotein W and NMR analysis of its interaction with 14-3-3 proteins. J. Biol. Chem..

[13] Kryukov G.V., Castellano S., Novoselov S.V., Lobanov A.V., Zehtab O., Guigo R., Gladyshev V.N. (2003). Characterization of mammalian selenoproteomes. Science.

[14] Fairweather-Tait S.J., Bao Y., Broadley M.R., Collings R., Ford D., Hesketh J.E., Hurst R. (2011). Selenium in human health and disease. Antioxid. Redox Signal..

[15] Rua R.M., Nogales F., Carreras O., Ojeda M.L. (2023). Selenium, selenoproteins and cancer of the thyroid. J.Trace Elem. Med. Biol..

[16] Detopoulou P., Letsiou S., Nomikos T., Karagiannis A., Pergantis S.A., Pitsavos C., Panagiotakos D.B., Antonopoulou S. (2023). Selenium, Selenoproteins and 10-year Cardiovascular Risk: Results from the ATTICA Study. Curr. Vasc. Pharmacol..

[17] Steinbrenner H., Duntas L.H., Rayman M.P. (2022). The role of selenium in type-2 diabetes mellitus and its metabolic comorbidities. Redox Biol..

[18] Lin Y., He F., Lian S., Xie B., Liu T., He J., Liu C. (2022). Selenium Status in Patients with Chronic Liver Disease: A Systematic Review and Meta-Analysis. Nutrients.

[19] Li Y., Chen D., Su J., Chen M., Chen T., Jia W., Zhu B. (2023). Selenium-ruthenium complex blocks H1N1 influenza virus-induced cell damage by activating GPx1/TrxR1. Theranostics.

[20] Ren Z., Okyere S.K., Zhang M., Zhang X., He H., Hu Y. (2022). Glycine Nano-Selenium Enhances Immunoglobulin and Cytokine Production in Mice Immunized with H9N2 Avian Influenza Virus Vaccine. Int. J. Mol. Sci..

[21] Guo M., Ye Y.-D., Cai J.-P., Xu H.-T., Wei W., Sun J.-Y., Wang C.-Y., Wang C.-B., Li Y.-H., Zhu B. (2024). PEG-SeNPs as therapeutic agents inhibiting apoptosis and inflammation of cells infected with H1N1 influenza A virus. Sci. Rep..

[22] Li Y., Lin Z., Guo M., Xia Y., Zhao M., Wang C., Xu T., Chen T., Zhu B. (2017). Inhibitory activity of selenium nanoparticles functionalized with oseltamivir on H1N1 influenza virus. Int. J. Nanomed..

[23] Li Y., Lin Z., Guo M., Zhao M., Xia Y., Wang C., Xu T., Zhu B. (2018). Inhibition of H1N1 influenza virus-induced apoptosis by functionalized selenium nanoparticles with amantadine through ROS-mediated AKT signaling pathways. Int. J. Nanomed..

[24] Zhang B., Liu M., Huang J., Zeng Q., Zhu Q., Xu S., Chen H. (2022). H1N1 Influenza A Virus Protein NS2 Inhibits Innate Immune Response by Targeting IRF7. Viruses.

[25] Chen Z., Zeng Y., Wei Y., Wang Q., Liu M., Zhang B., Liu J., Zhu Q., Xu S. (2022). Influenza D virus Matrix protein 1 restricts the type I interferon response by degrading TRAF6. Virology.

[26] Yuan H. (2021). Construction and Visualization Research of Influenza Fluorescence Reporter Virus. Master’s Thesis.

[27] Zhang B., Xu S., Liu M., Wei Y., Wang Q., Shen W., Lei C.Q., Zhu Q. (2023). The nucleoprotein of influenza A virus inhibits the innate immune response by inducing mitophagy. Autophagy.

[28] Wang S., Liang Z., Gong Y., Yin Y., Wang K., He Q., Wang Z., Bai J. (2016). Confocal ramanmicrospectral imaging of ex vivo human spinal cord tissue. J. Photochem. Photobiol. B.

[29] Chida J., Hara H., Yano M., Uchiyama K., Das N.R., Takahashi E., Miyata H., Tomioka Y., Ito T., Kido H. (2018). Prion protein protects mice from lethal infection with influenza A viruses. PLoS Pathog..

[30] Reeves M.A., Bellinger F.P., Berry M.J. (2010). The neuroprotective functions of selenoprotein M and its role in cytosolic calcium regulation. Antioxid. Redox Signal..

[31] Korotkov K.V., Novoselov S.V., Hatfield D.L., Gladysgev V.N. (2002). Mammalian selenopritein in which selenocysteine (Sec) incorporation is supported by a new form of Sec insertion sequence element. Mol. Cell. Biol..

[32] Hwang D.Y., Sin J.S., Kim M.S., Yim S.Y., Kim Y.K., Kim C.K., Kim B.G., Shim S.B., Jee S.W., Lee S.H. (2008). Overexpression of human selenoprotein M differentially regulates the concentrations of antioxidant and H_2_O_2_, the activity of antioxidant enzymes, and the compositions of white blood cells in a transgenic rat. Int. J. Mol. Med..

[33] Loef M., Schrauzer G.N., Walach H. (2011). Selenium and alzheimer’s disease: A systematic review. J. Alzheimer’s Dis..

[34] Wheeler J.L., Martin K.C., Lawrence B.P. (2013). Novel cellular targets of AhR underlie alterations in neutrophilic inflammation and inducible nitric oxide synthase expression during influenza virus infection. J. Immunol..

[35] Vlahos R., Stambas J., Selemidis S. (2012). Suppressing production of reactive oxygen species (ROS) for influenza A virus therapy. Trends Pharmacol. Sci..

[36] To E.E., Erlich J.R., Liong F., Luong R., Liong S., Esaq F., Oseghale O., Anthony D., McQualter J., Bozinovski S. (2020). Mitochondrial Reactive Oxygen Species Contribute to Pathological Inflammation During Influenza A Virus Infection in Mice. Antioxid. Redox Signal..

[37] Head J.L., Lawrence B.P. (2009). The aryl hydrocarbon receptor is a modulator of anti-viral immunity. Biochem. Pharmacol..

[38] Data Center for Public Health Sciences, Chinese Center for Disease Control and Prevention. https://www.phsciencedata.cn.

