# Correction: Liu et al. Selenoprotein M Inhibits the Replication of Influenza A Virus by Regulating Reactive Oxygen Species Levels. *Life* 2025, *15*, 714

**DOI:** 10.3390/life15060886

**Published:** 2025-05-30

**Authors:** Minxuan Liu, Jinhui Wang, Weigang Li, Bo Zhao, Yuanyuan Zhang, Qiaoying Zeng, Guangyuan Liu

**Affiliations:** 1 College of Veterinary Medicine, Gansu Agricultural University, Lanzhou 730070, China; mxliu1991@163.com (M.L.); wangjinhui0606@163.com (J.W.); 2 State Key Laboratory of Veterinary Etiological Biology, Key Laboratory of Veterinary Parasitology of Gansu Province, Lanzhou Veterinary Research Institute, Chinese Academy of Agricultural Science, Lanzhou 730046, China; 3 Animal Husbandry and Veterinary Station of Zhenyuan County, Zhenyuan 744500, China; 18719718116@163.com (W.L.); 18294092571@163.com (Y.Z.); 4 Gansu Agriculture Technology College, Duanjiatan 425, Lanzhou 730030, China; zbtaobaozy@gmail.com

## Corresponding Author Sequence Corrected

In the original publication [1], there was an error regarding the sequence of the corresponding authors Guangyuan Liu and Qiaoying Zeng. The corrected sequence of the corresponding authors is Qiaoying Zeng and Guangyuan Liu.

The authors state that the scientific conclusions are unaffected. This correction was approved by the Academic Editor. The original publication has also been updated.
