# Supplementary material for: Selenoprotein M Inhibits the Replication of Influenza A Virus by Regulating Reactive Oxygen Species Levels
Source: Life (Basel). 2025 Apr 28;15(5):714. doi: 10.3390/life15050714 (PMC12112756; doi:10.3390/life15050714)
Supplement: Supplementary file 1 [file life-15-00714-s001.zip › si-RNA sequences report -P202010090070-RIBOBIO.pdf]

RIBOBIO

序列报告 – P202010090070

产品编号	产品名称	靶序列
stB0003784A	genOFFTM st-h-GPX1_001	GCAAGGTACTACTTATCGA
stB0006519A	genOFFTM st-h-GPX2_001	CCAAAAATGTGAGGTGAAT
stB0006520A	genOFFTM st-h-GPX3_001	CCATGAAGGTTCACGACAT
stB0003785A	genOFFTM st-h-GPX4_001	GCTACAACGTCAAATTCGA
stB0016911A	genOFFTM st-h-GPX6_001	GCAAGCACGTCCTGTTTGT
stB0006141A	genOFFTM st-h-DIO1_001	GAGTCAAGCGGAACATCCT
stB0006142A	genOFFTM st-h-DIO2_001	TCCTCGATGCCTACAAACA
stB0006143A	genOFFTM st-h-DIO3_001	CATCCAGAGTGGCACTATT
stB0008210A	genOFFTM st-h-TXNRD1_001	GGACGATTCCGTCAAGAGA
stB0014932A	genOFFTM st-h-TXNRD3_001	GGAGAAGATTGGTGTCAAA
stB0009600A	genOFFTM st-h-TXNRD2_001	GCATCCCAGTGTTATGTAA
stB0011455A	genOFFTM st-h-MSRB1_001	GGAGCACAATAGATCTGAA
stB0010014A	genOFFTM st-h-SEPHS2_001	GGTCAACCCTTGGATTATA
stB0007823A	genOFFTM st-h-SEPW1_001	GCAAGTTTCTGAAGTTGGT
stB0018814A	genOFFTM st-h-SELT_001	GTAGAAGAGTAGTCATGTA
stB0016945A	genOFFTM st-h-C11orf31_001	GACCGTTGTTATCGAGCAT
stB0018815A	genOFFTM st-h-SELV_001	GGAGACTGGTCCATTCCAA
stB0014501A	genOFFTM st-h-EPT1_001	GTATGAACCTTTCCTGTTT
stB0019128A	genOFFTM st-h-SEP15_001	GATCCTGATTGCAGAGGAT
stB0018823A	genOFFTM st-h-SELM_001	GCATCCCACTCAGTGAAAT
stB0018822A	genOFFTM st-h-SELK_001	GTGGAAGGCTGACCATGAT
stB0012272A	genOFFTM st-h-VIMP_001	GGACAGCATGCAAGAAGGA
stB0018824A	genOFFTM st-h-SELO_001	GCAGAACTGTGCGTGACAT
stB0012566A	genOFFTM st-h-SEPN1_001	CCGAGGAAATCGAGAGCAA
stB0007822A	genOFFTM st-h-SEPP1_001	GTGGCCGTCTTGTATATCA

所有 siRNA 对照、ASO 对照、Smart Silencer 对照的序列信息都不公开，如需引用，请指明出处，不会影响文章的发表。感谢您对锐博生物的支持，祝您一切顺利！

GUANGZHOU RIBOBIO CO., LTD.
13-14F, Innovation Bldg C3, 182 Kexue Ave, Science Park, Guangzhou 510663, China
Hot line: 400 686 0075 Tel: +86 (20) 3229 0075 Web: www.ribobio.com Email: marketing@ribobio.com
