# Supplementary figures and images for: Selenoprotein M Inhibits the Replication of Influenza A Virus by Regulating Reactive Oxygen Species Levels
Source: Life (Basel). 2025 Apr 28;15(5):714. doi: 10.3390/life15050714 (PMC12112756; doi:10.3390/life15050714)

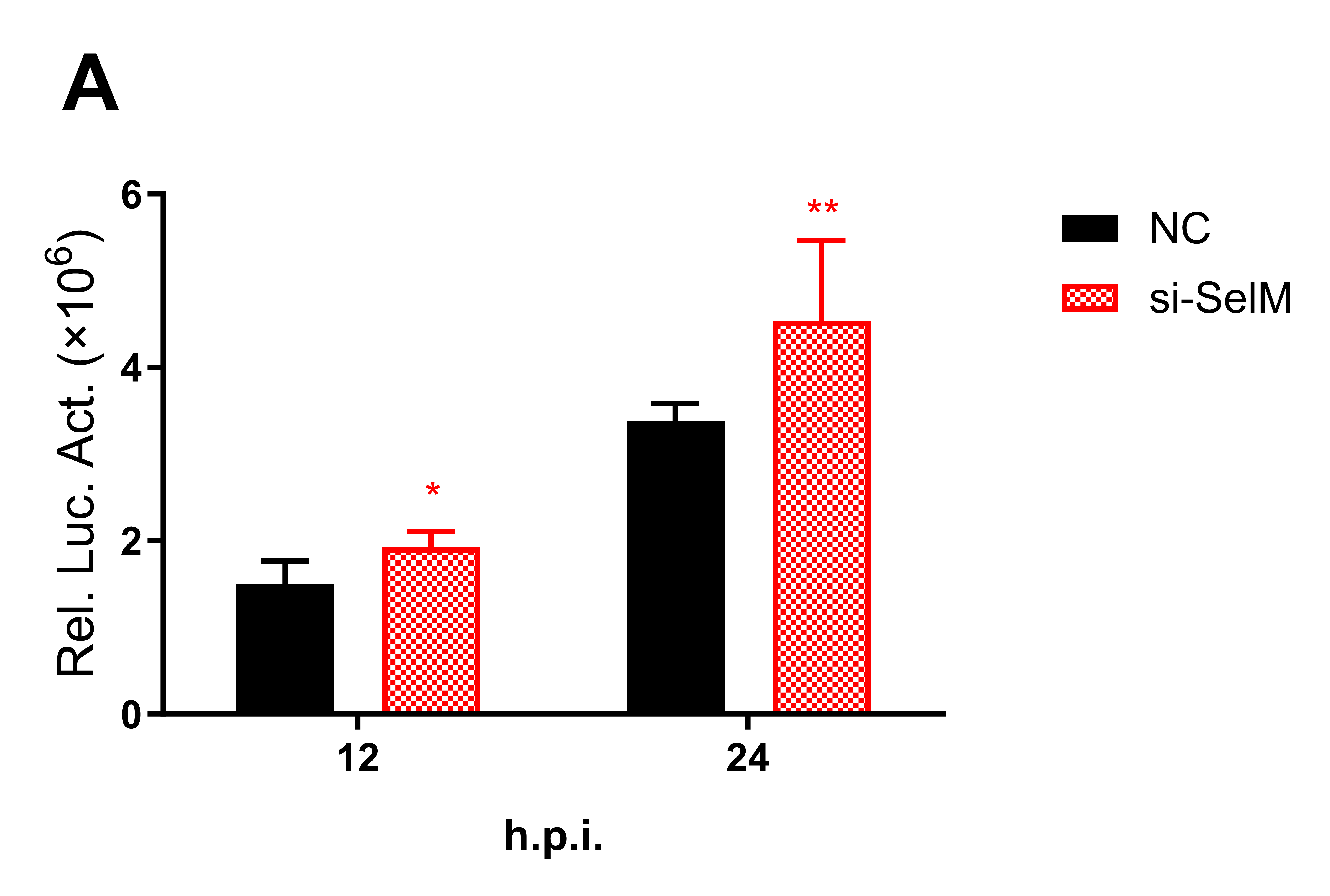

Supplement: Supplementary file 1 [file life-15-00714-s001.zip › Fig.1/Fig.1-A.tif]

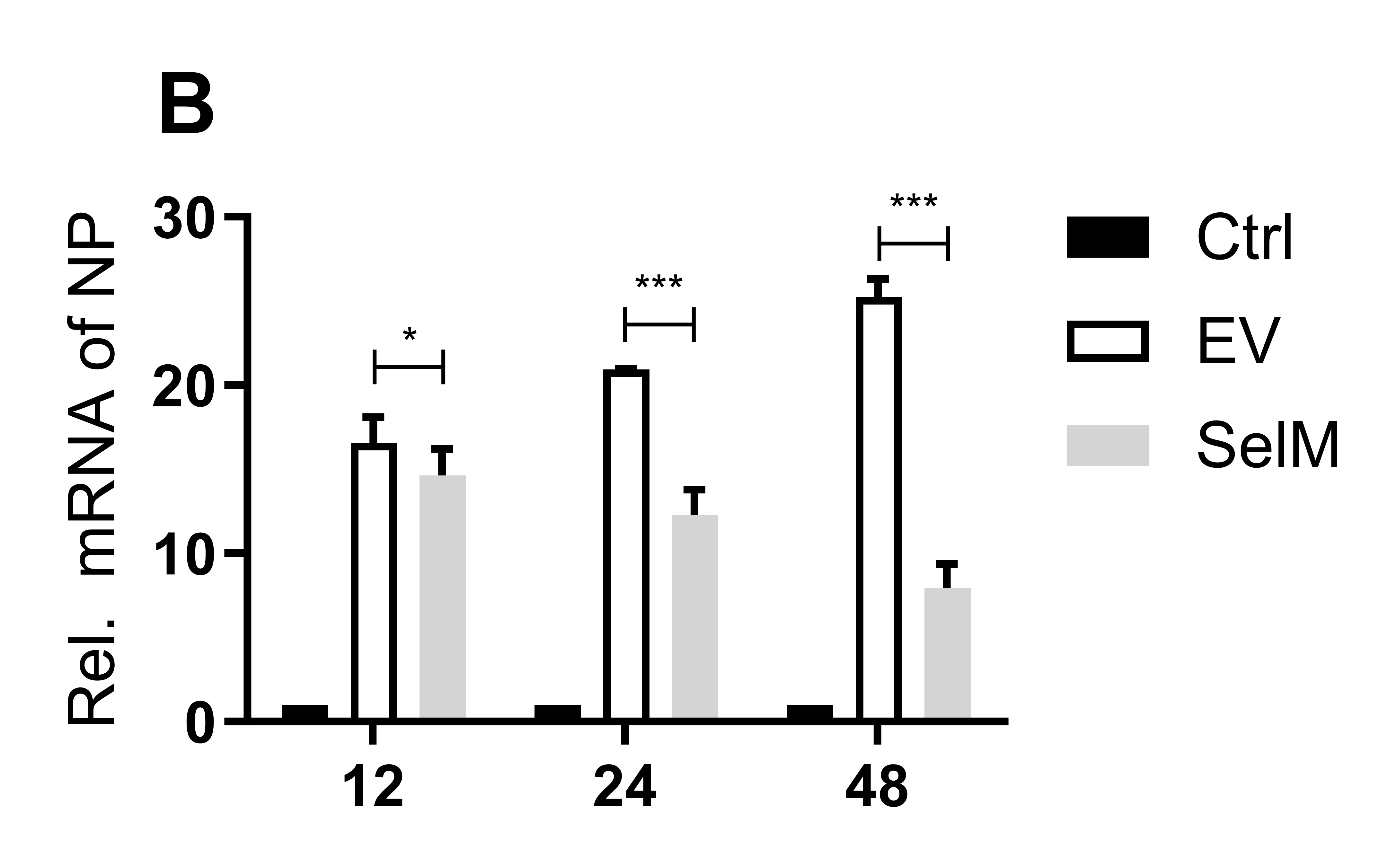

Supplement: Supplementary file 1 [file life-15-00714-s001.zip › Fig.1/Fig.1-B.tif]

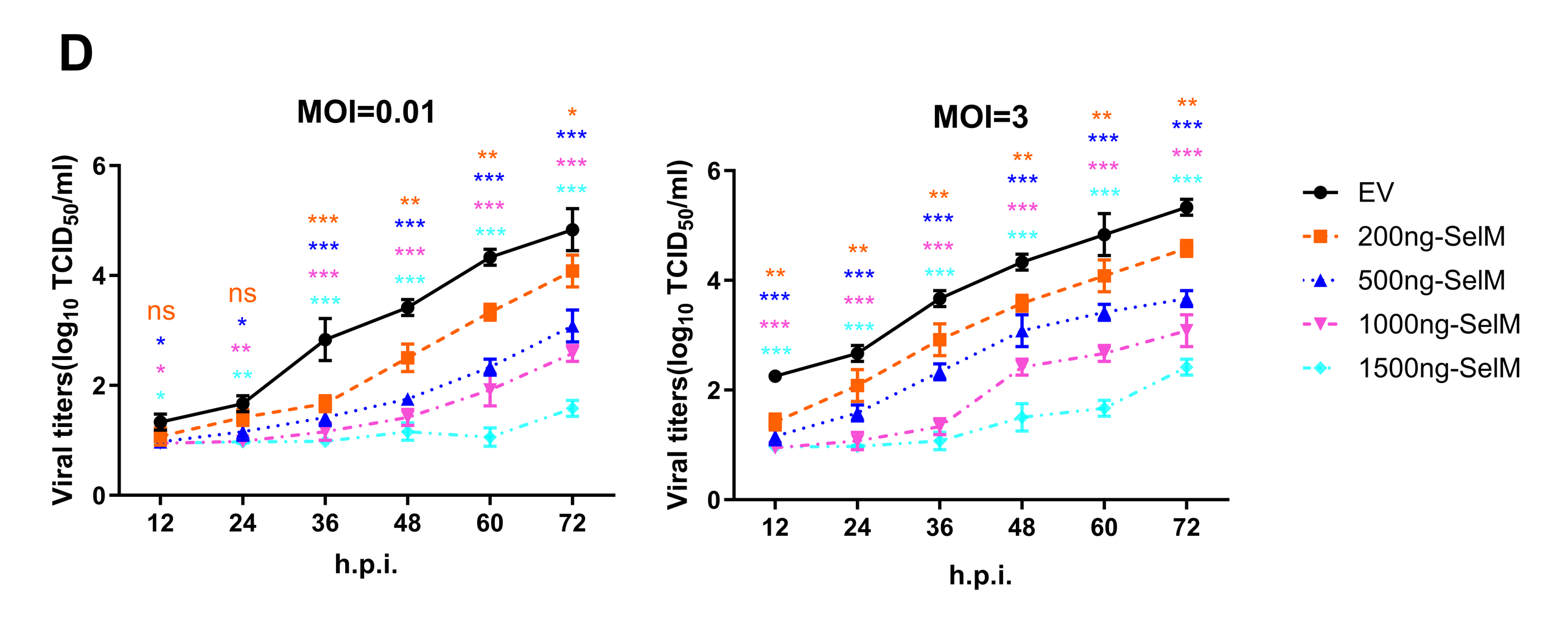

Supplement: Supplementary file 1 [file life-15-00714-s001.zip › Fig.1/Fig.1-D.tif]

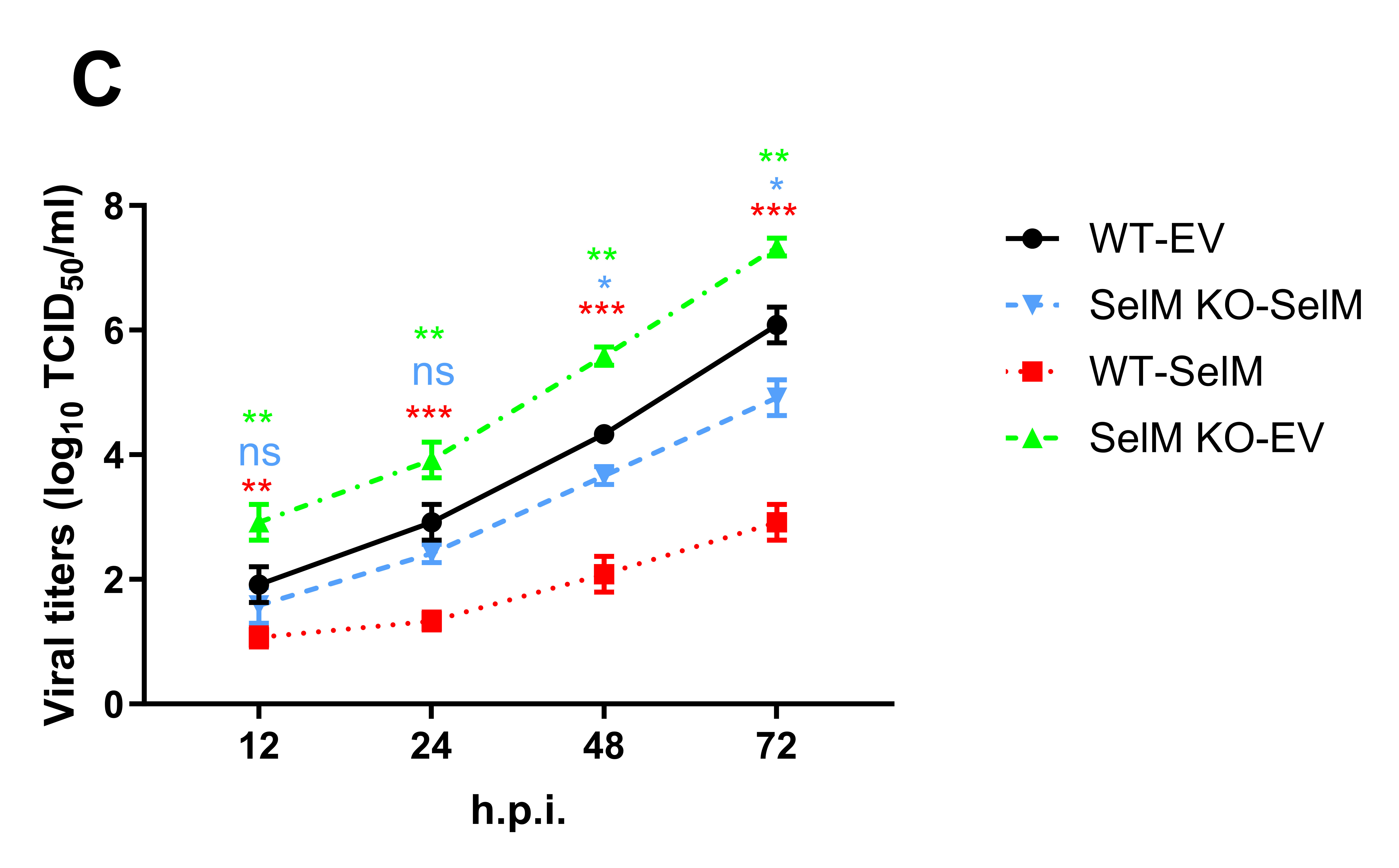

Supplement: Supplementary file 1 [file life-15-00714-s001.zip › Fig.2/Fig,2-C.tif]

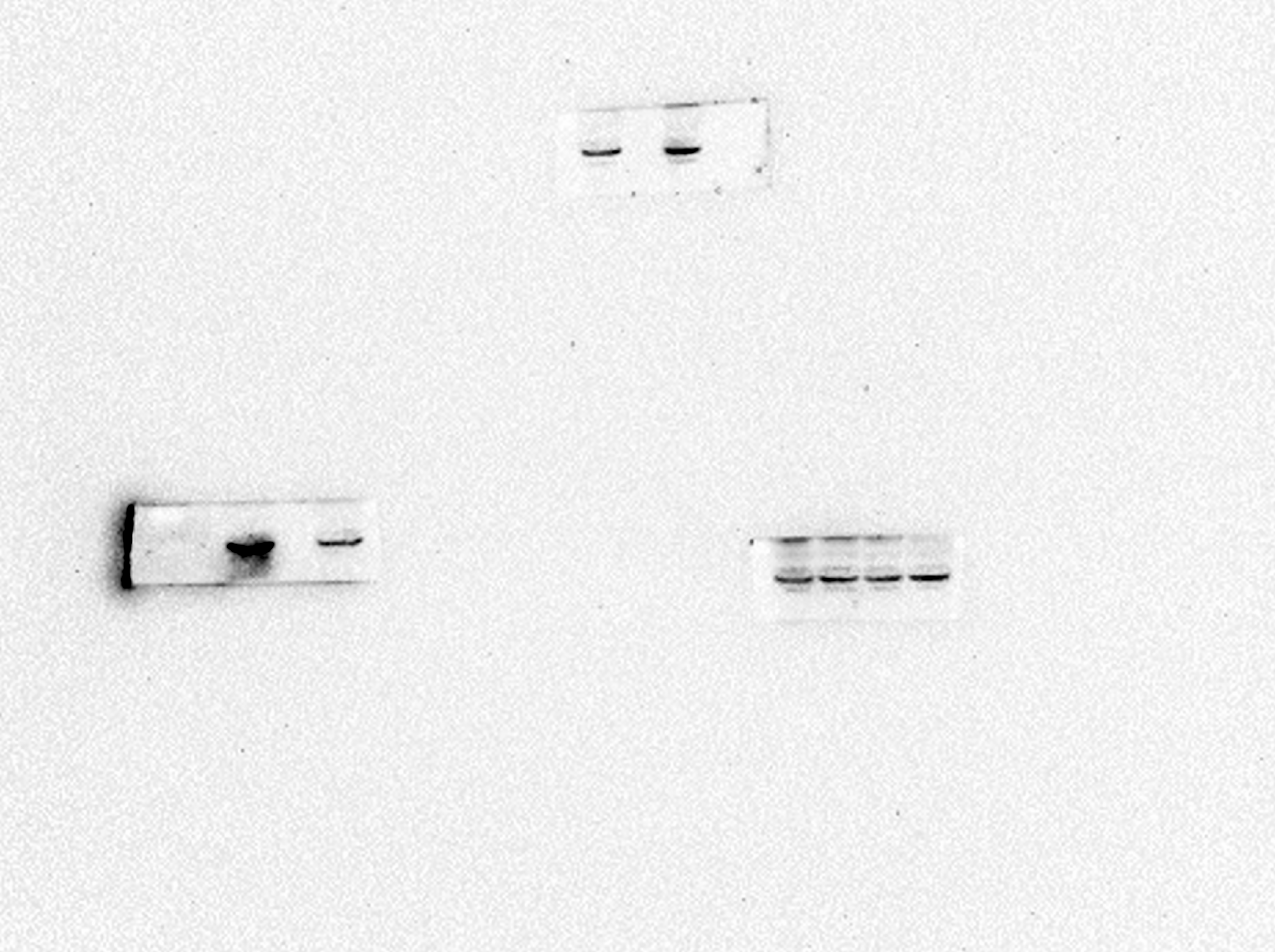

Supplement: Supplementary file 1 [file life-15-00714-s001.zip › Fig.2/Fig.2-A/2-2 (Chemi).tif]

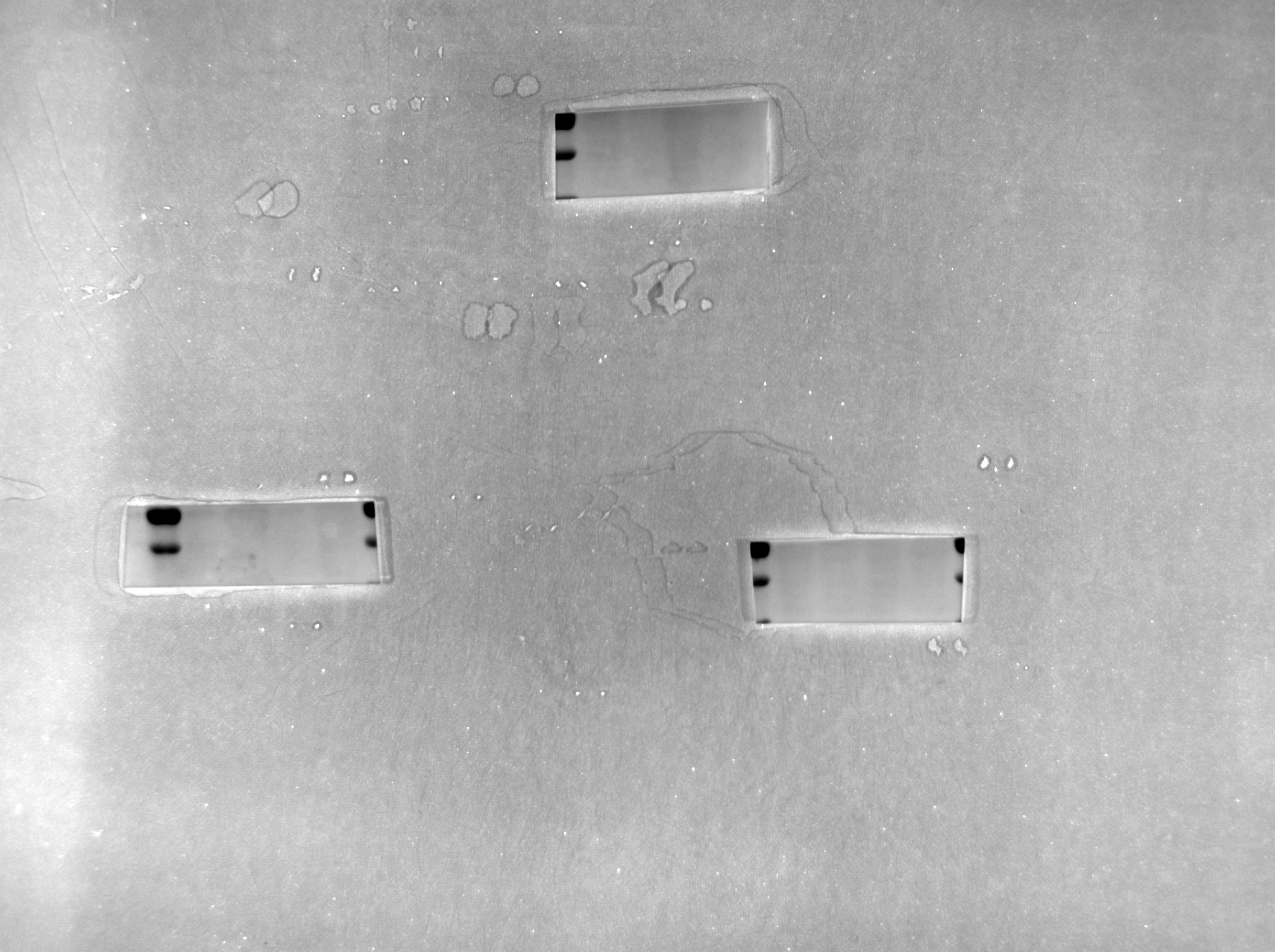

Supplement: Supplementary file 1 [file life-15-00714-s001.zip › Fig.2/Fig.2-A/2-2 (DyLight 488).tif]

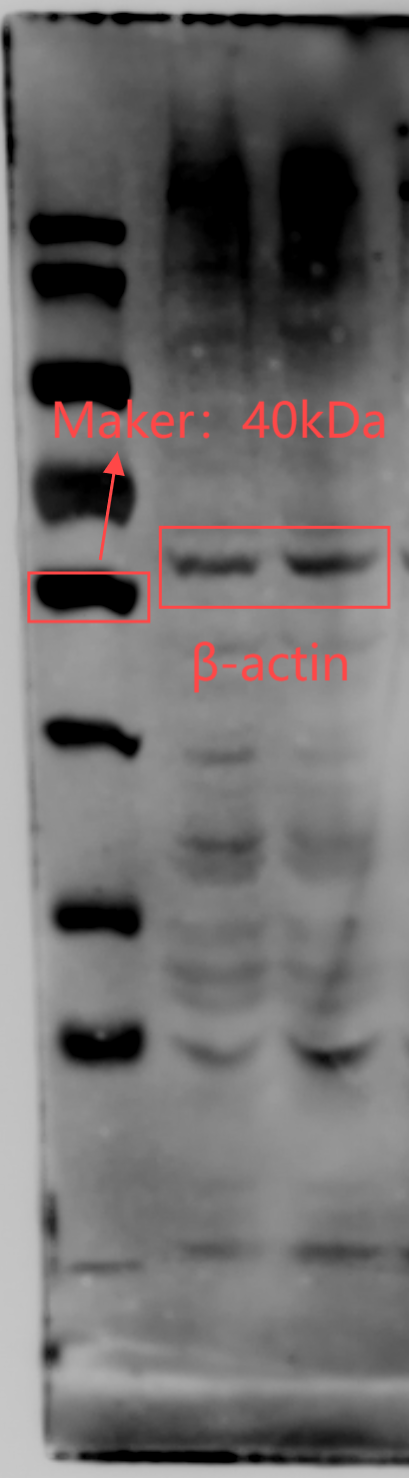

Supplement: Supplementary file 1 [file life-15-00714-s001.zip › Fig.2/Fig.2-A/ANTI-β-actin-Figure legend.png]

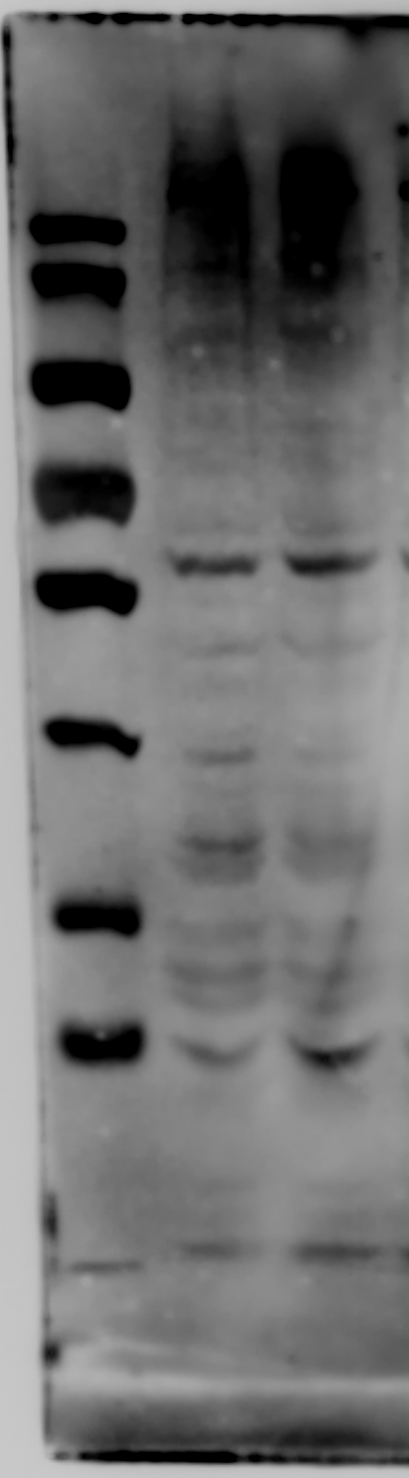

Supplement: Supplementary file 1 [file life-15-00714-s001.zip › Fig.2/Fig.2-A/ANTI-β-actin.png]

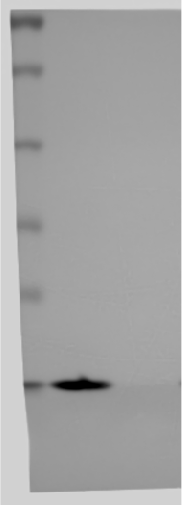

Supplement: Supplementary file 1 [file life-15-00714-s001.zip › Fig.2/Fig.2-A/Experimental replicates-Figure legend(1).png]

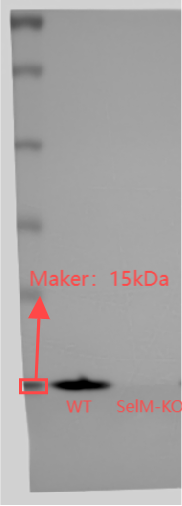

Supplement: Supplementary file 1 [file life-15-00714-s001.zip › Fig.2/Fig.2-A/Experimental replicates-Figure legend.png]

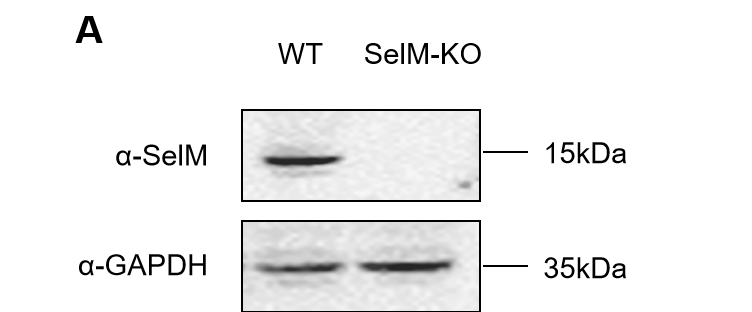

Supplement: Supplementary file 1 [file life-15-00714-s001.zip › Fig.2/Fig.2-A/Fig.2-A.tif]

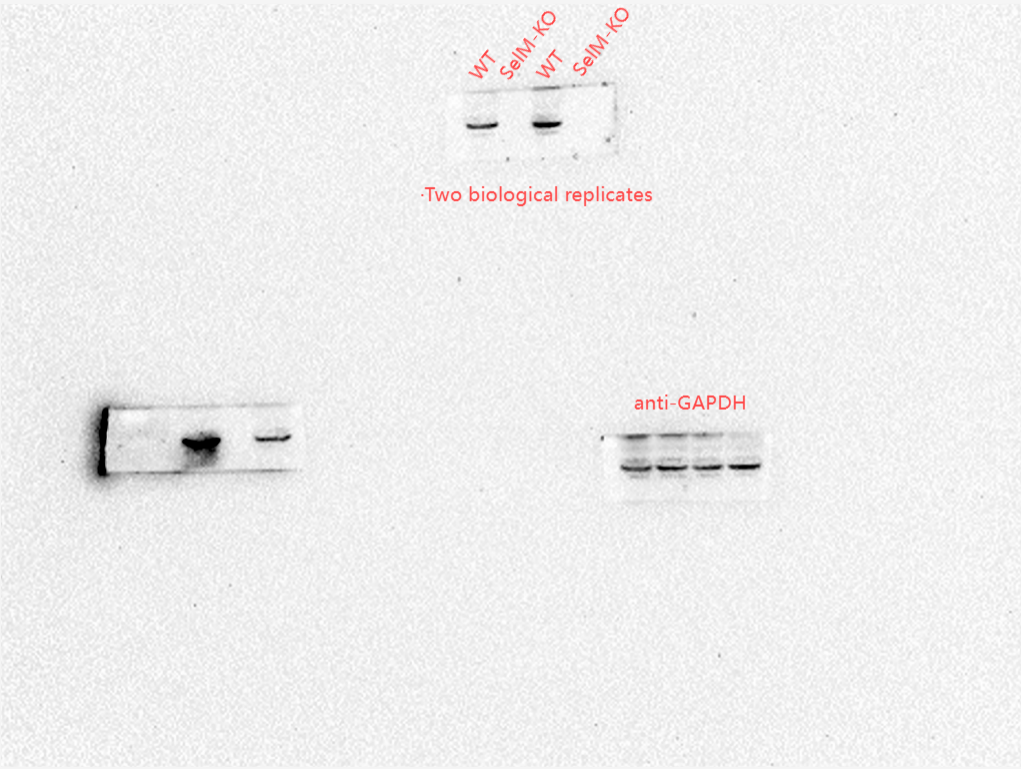

Supplement: Supplementary file 1 [file life-15-00714-s001.zip › Fig.2/Fig.2-A/Figure legend.png]

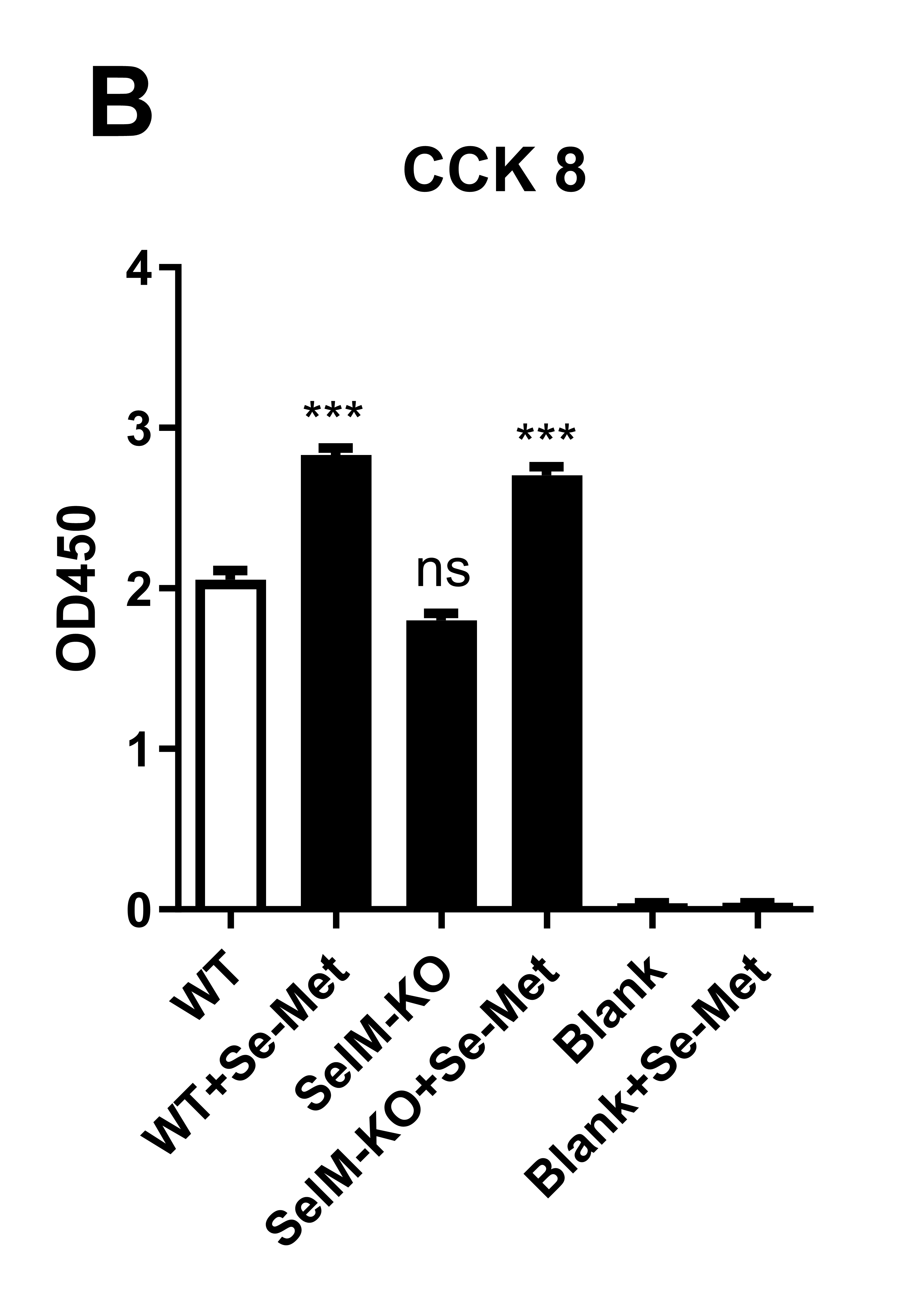

Supplement: Supplementary file 1 [file life-15-00714-s001.zip › Fig.2/Fig.2-B.tif]

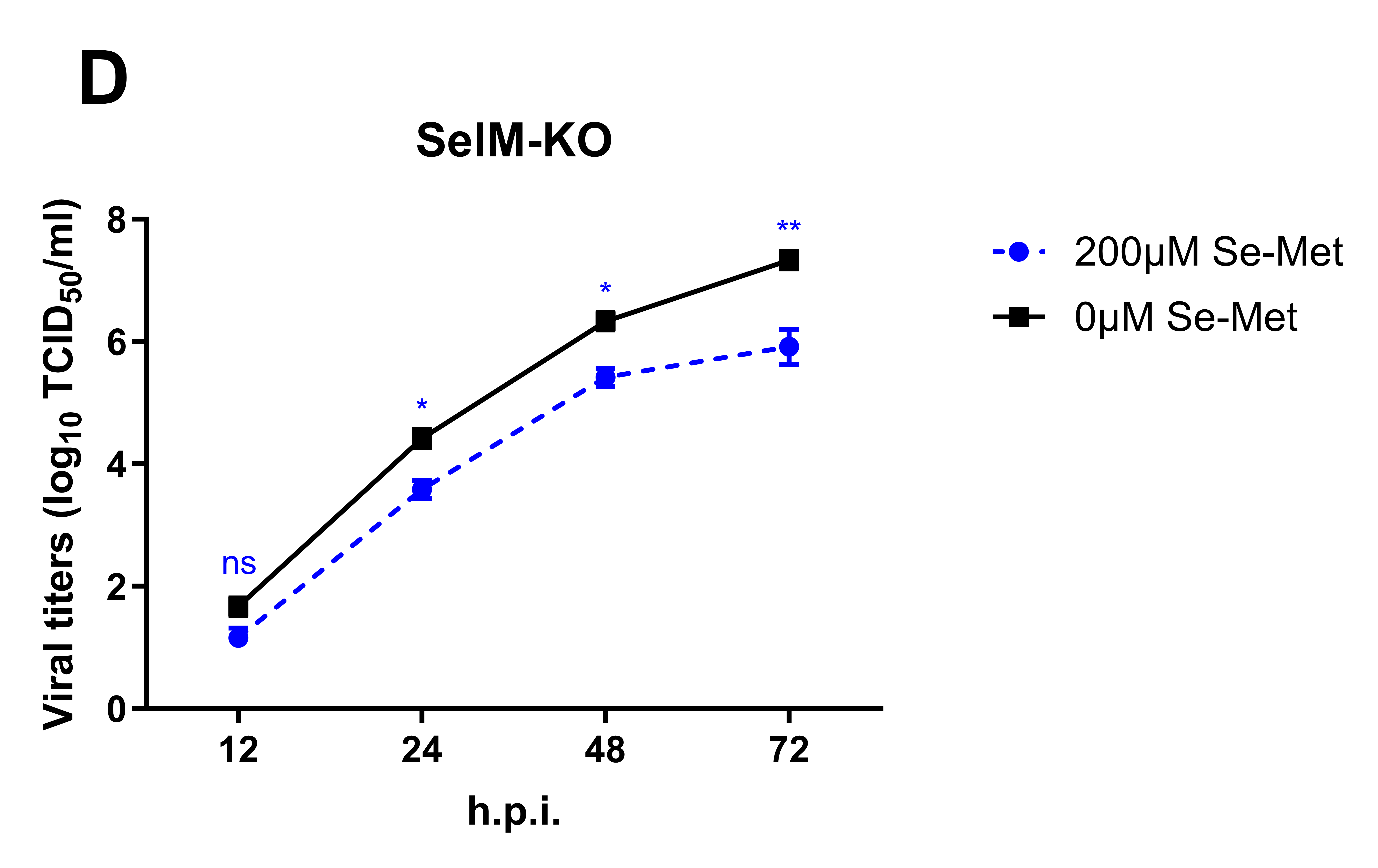

Supplement: Supplementary file 1 [file life-15-00714-s001.zip › Fig.2/Fig.2-D.tif]

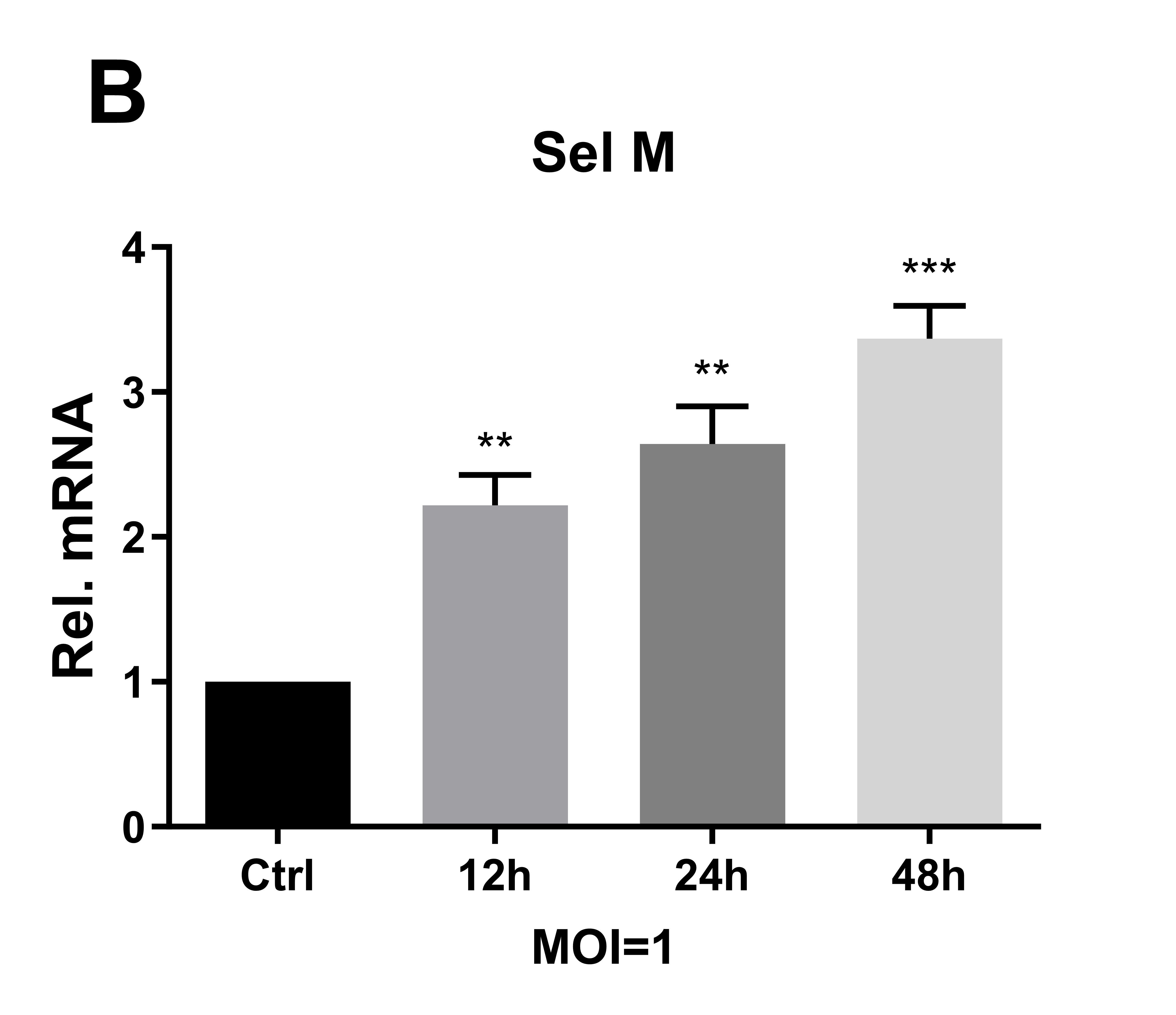

Supplement: Supplementary file 1 [file life-15-00714-s001.zip › Fig.3/Fig,3-B.tif]

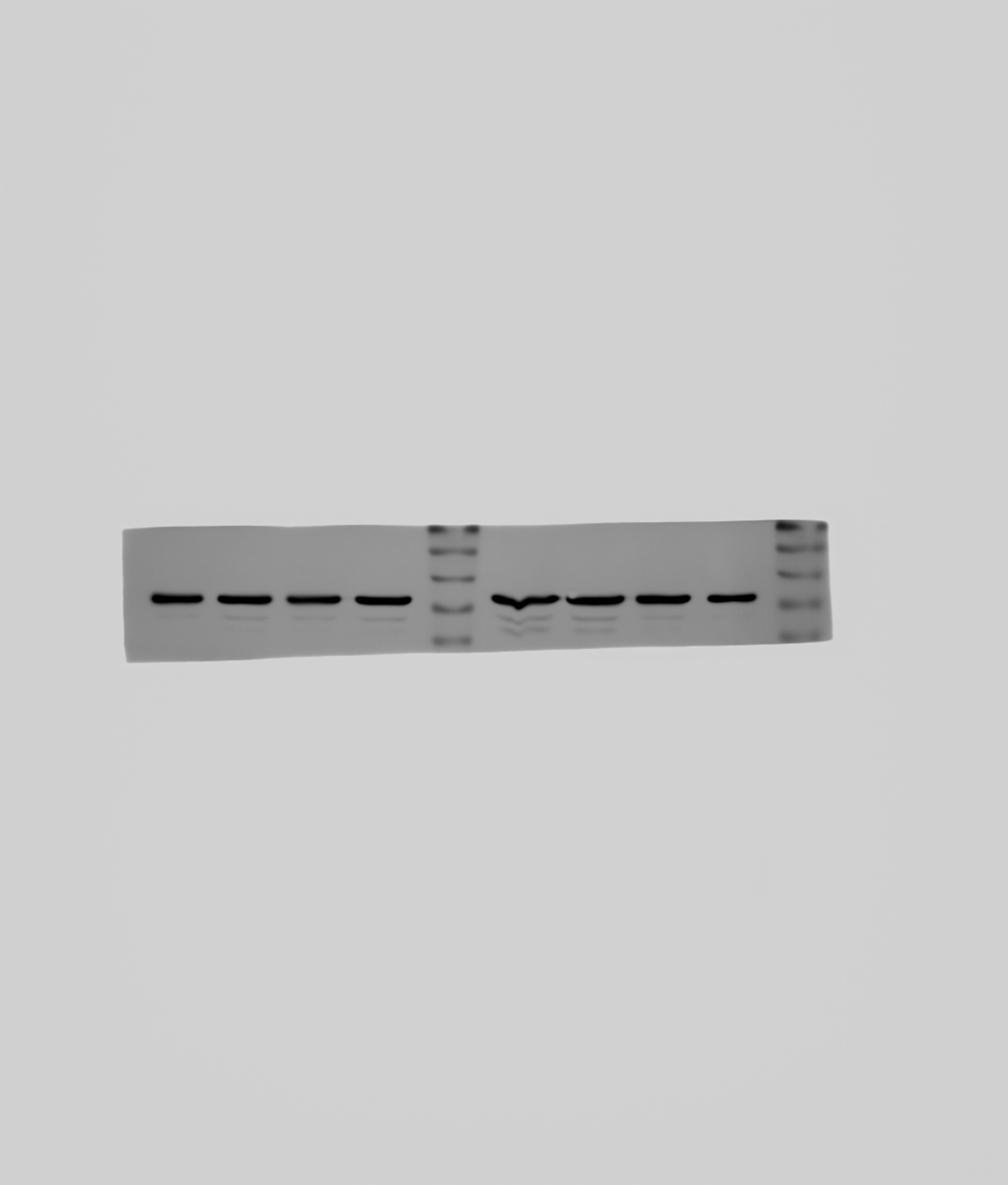

Supplement: Supplementary file 1 [file life-15-00714-s001.zip › Fig.3/fig.3-A/3A-GAPDH .tif]

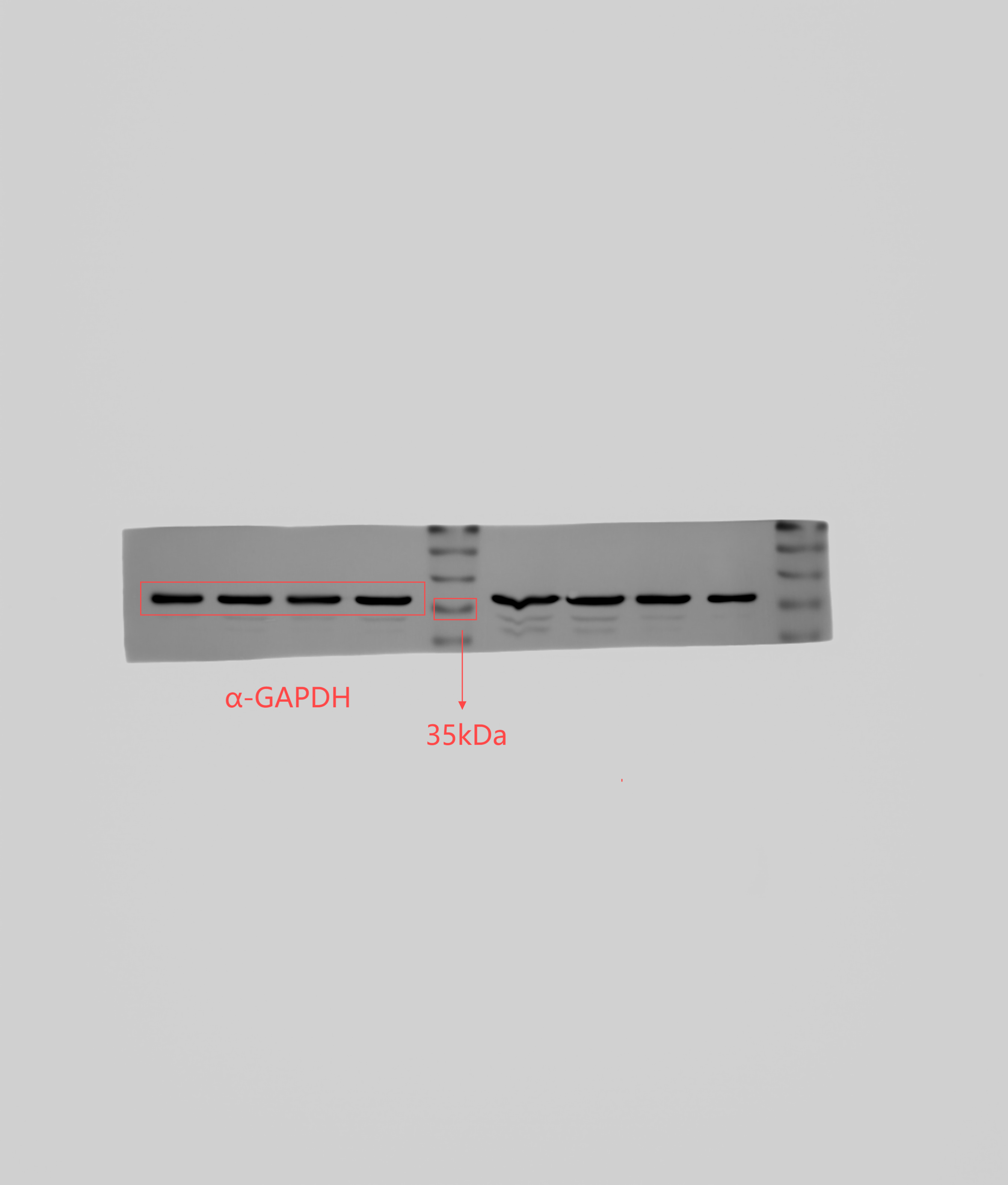

Supplement: Supplementary file 1 [file life-15-00714-s001.zip › Fig.3/fig.3-A/3A-GAPDH-Figure legend.png]

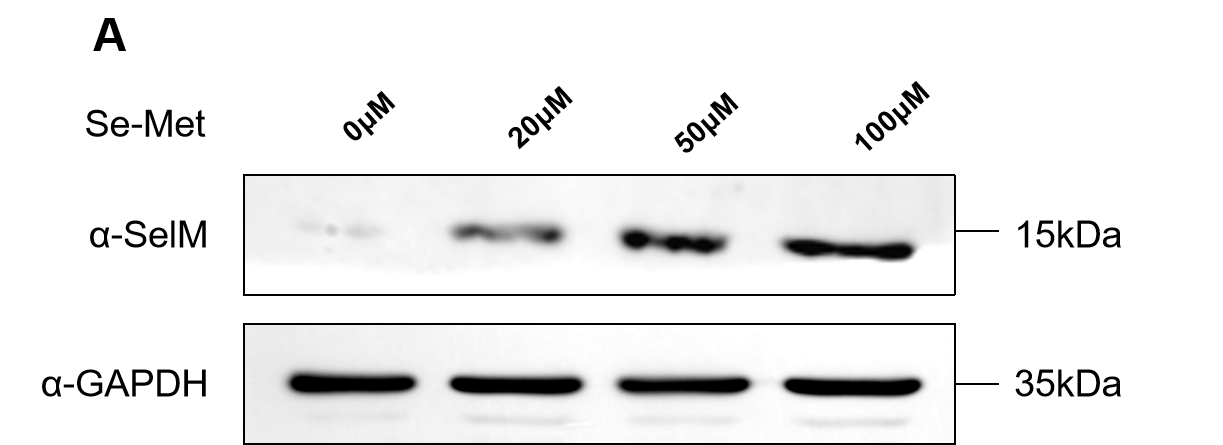

Supplement: Supplementary file 1 [file life-15-00714-s001.zip › Fig.3/fig.3-A/Fig.3-A.tif]

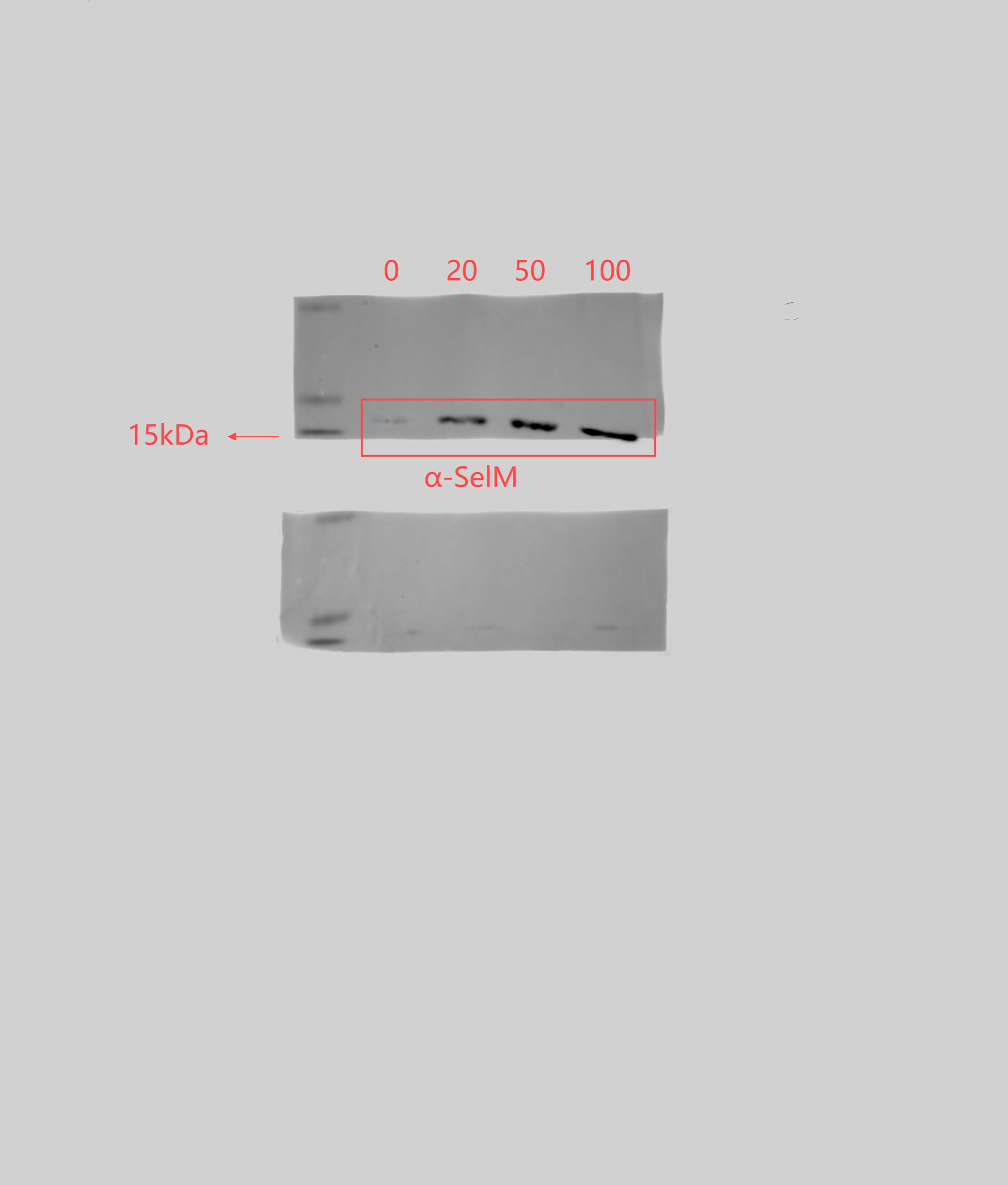

Supplement: Supplementary file 1 [file life-15-00714-s001.zip › Fig.3/fig.3-A/fig.3A.png]

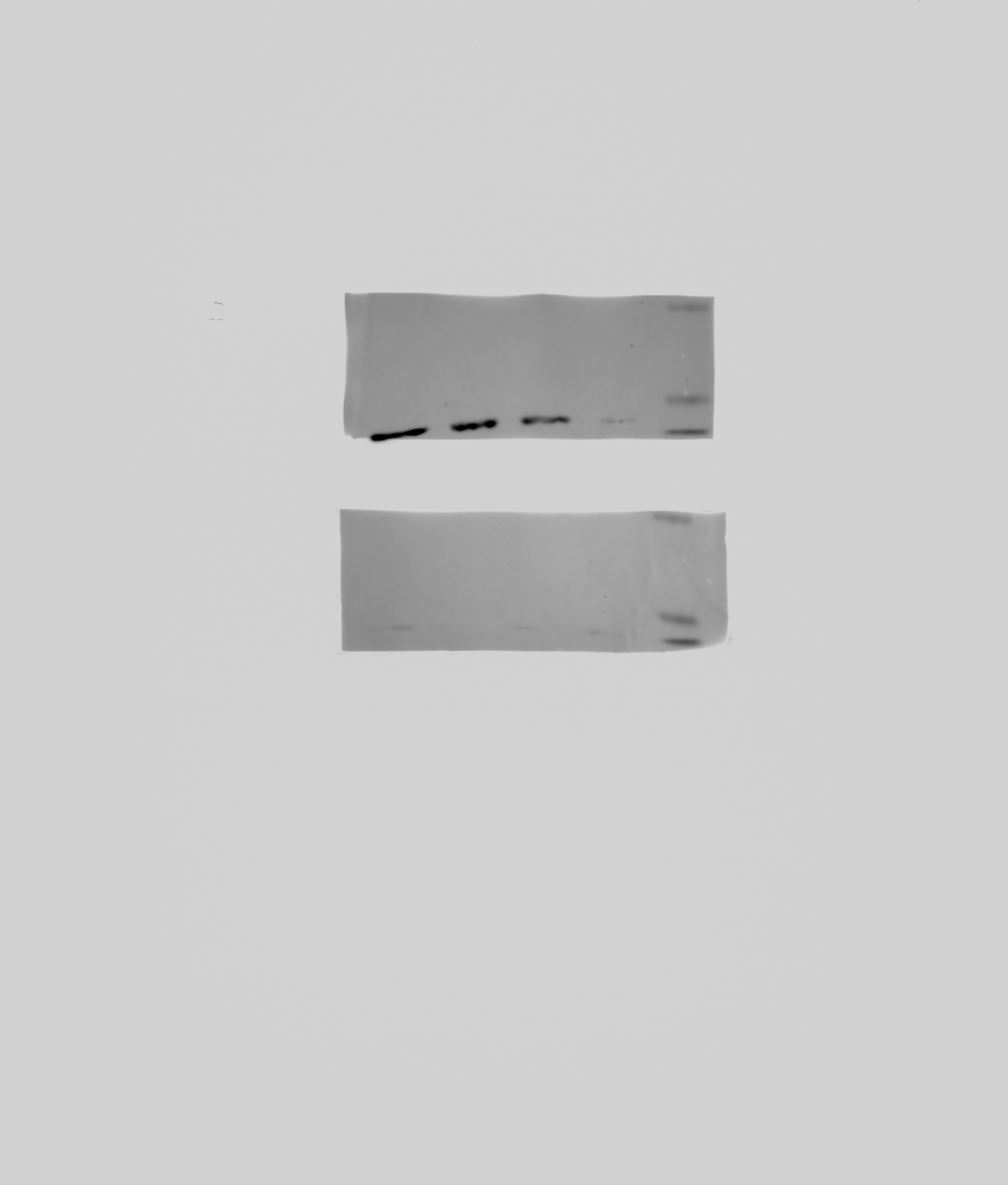

Supplement: Supplementary file 1 [file life-15-00714-s001.zip › Fig.3/fig.3-A/fig.3A.tif]

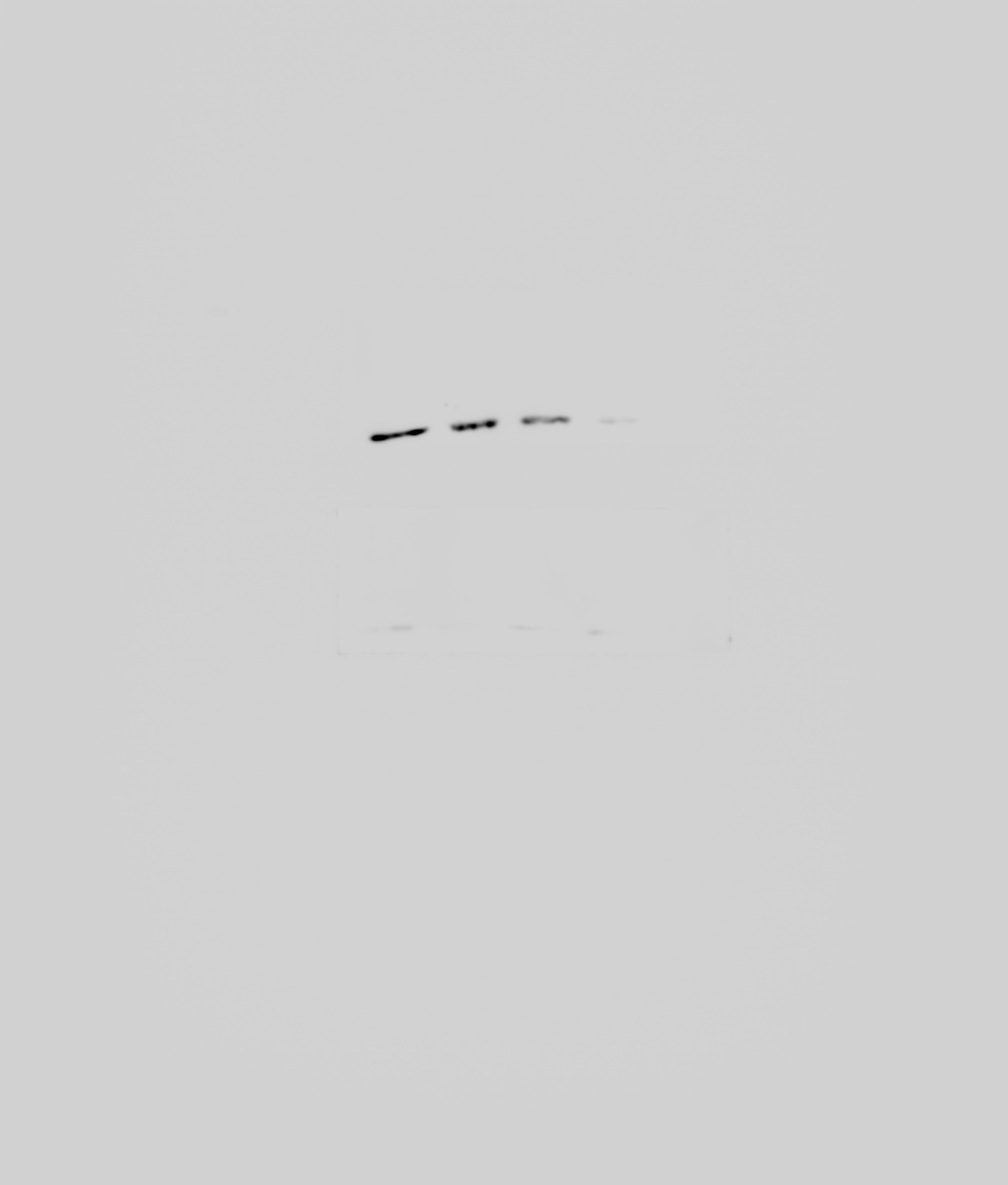

Supplement: Supplementary file 1 [file life-15-00714-s001.zip › Fig.3/fig.3-A/SelM-sample.tif]

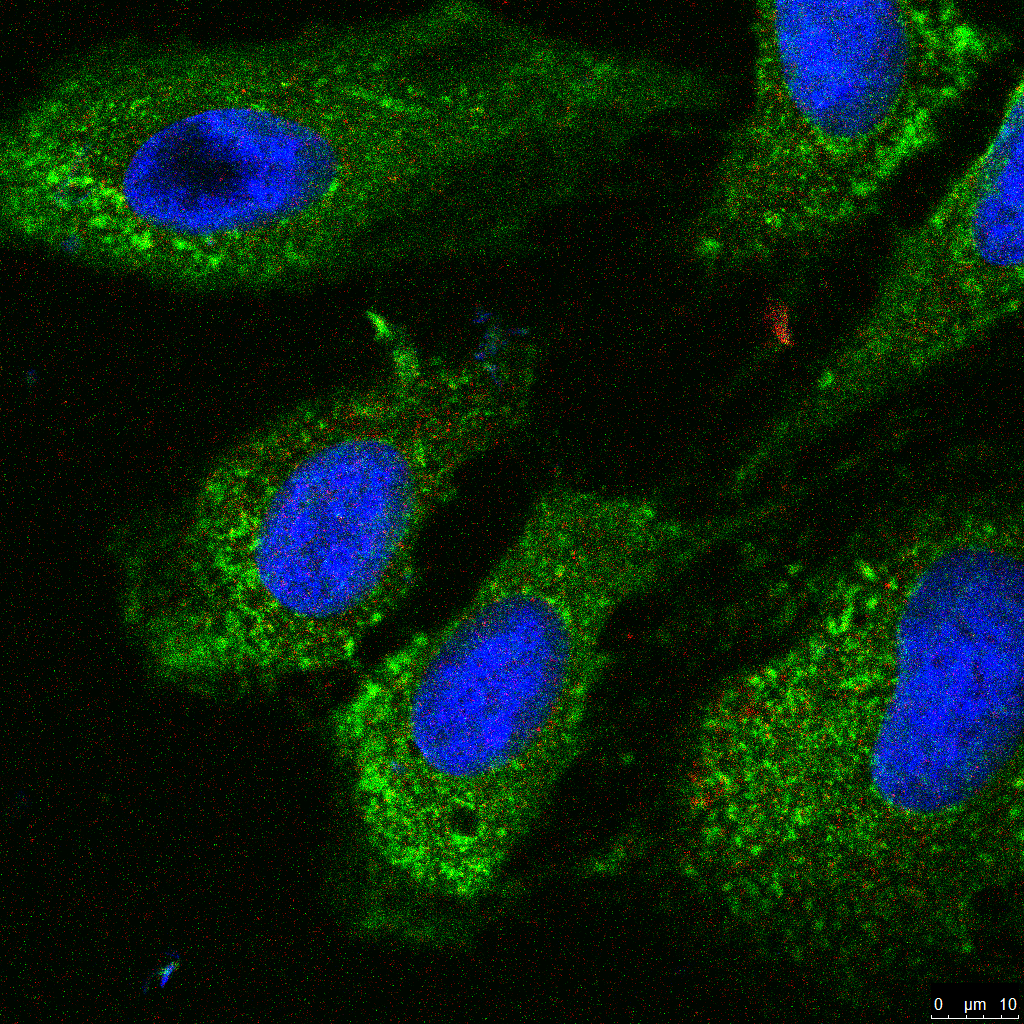

Supplement: Supplementary file 1 [file life-15-00714-s001.zip › Fig.3/fig.3-C/Experiment_EV-12_z0.tif]

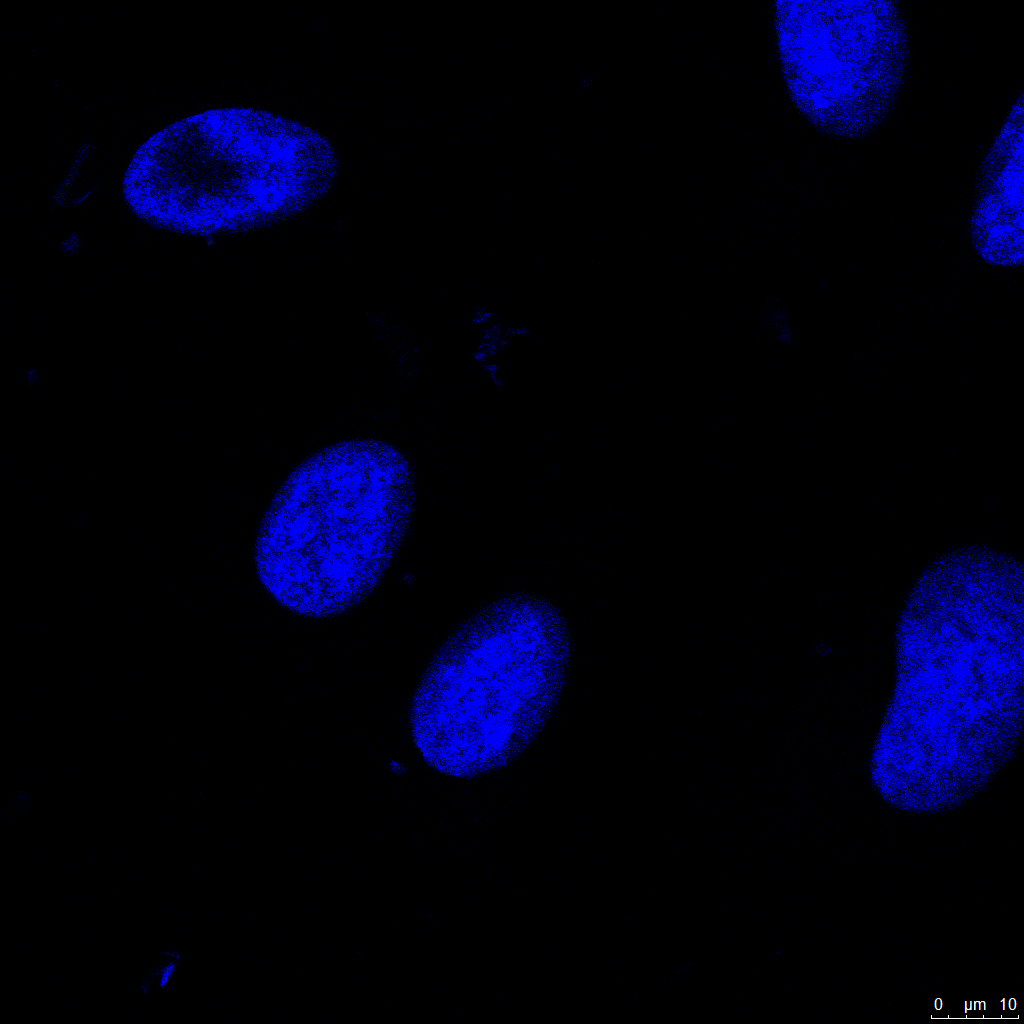

Supplement: Supplementary file 1 [file life-15-00714-s001.zip › Fig.3/fig.3-C/Experiment_EV-12_z0_ch00.tif]

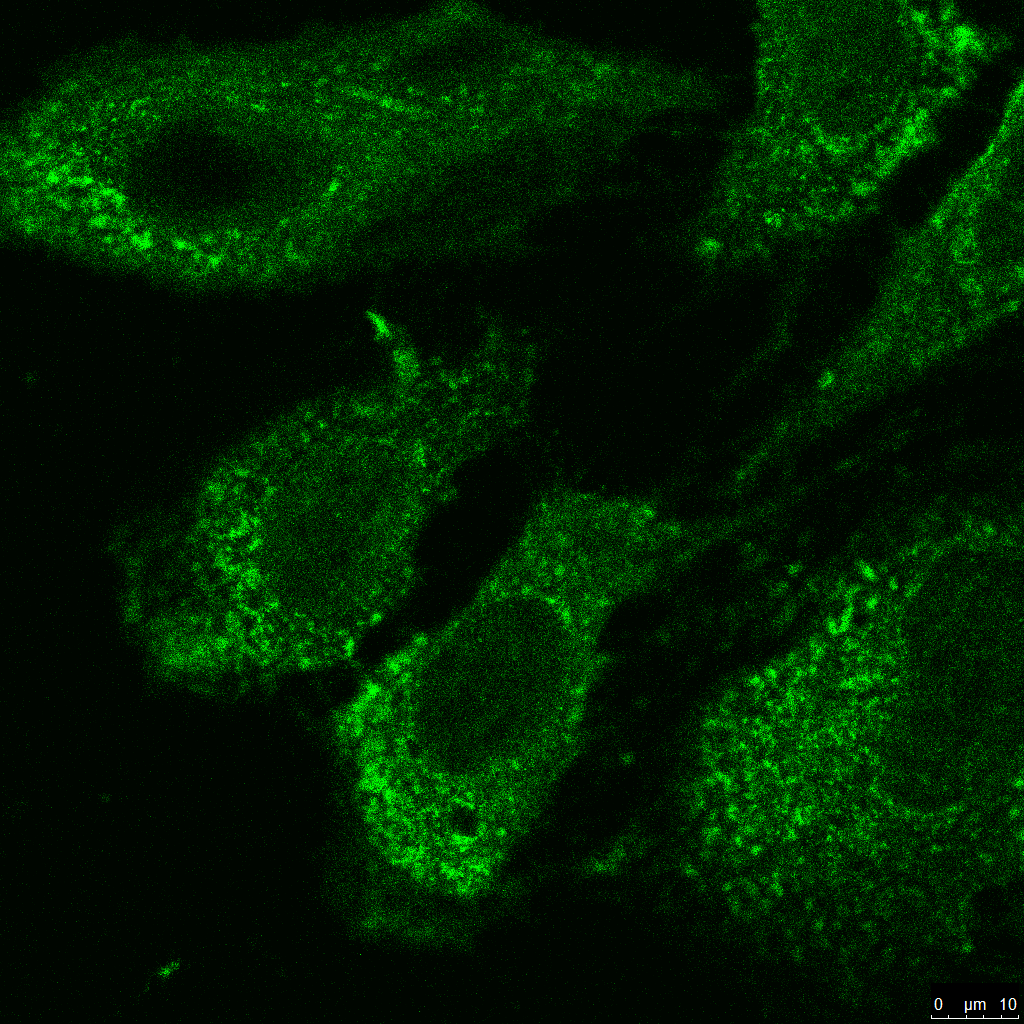

Supplement: Supplementary file 1 [file life-15-00714-s001.zip › Fig.3/fig.3-C/Experiment_EV-12_z0_ch01.tif]

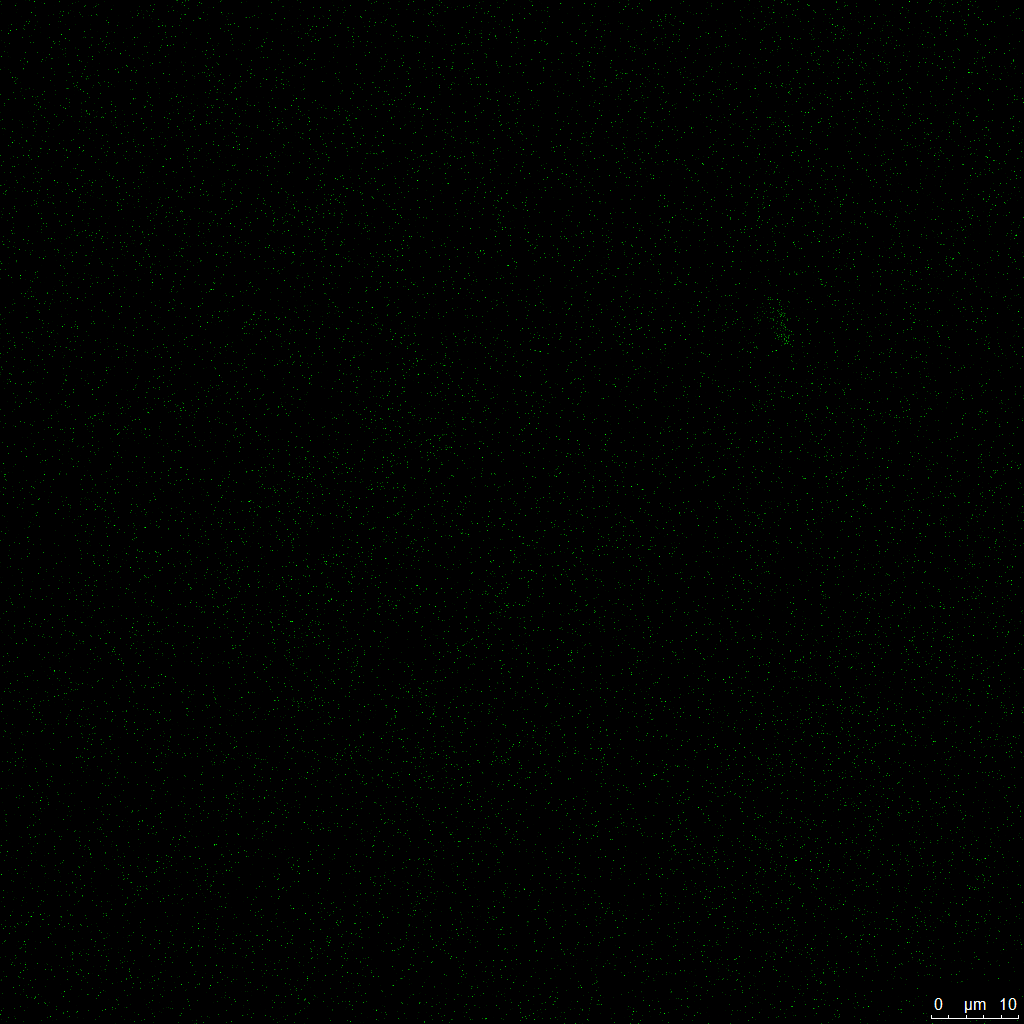

Supplement: Supplementary file 1 [file life-15-00714-s001.zip › Fig.3/fig.3-C/Experiment_EV-12_z0_ch03.tif]

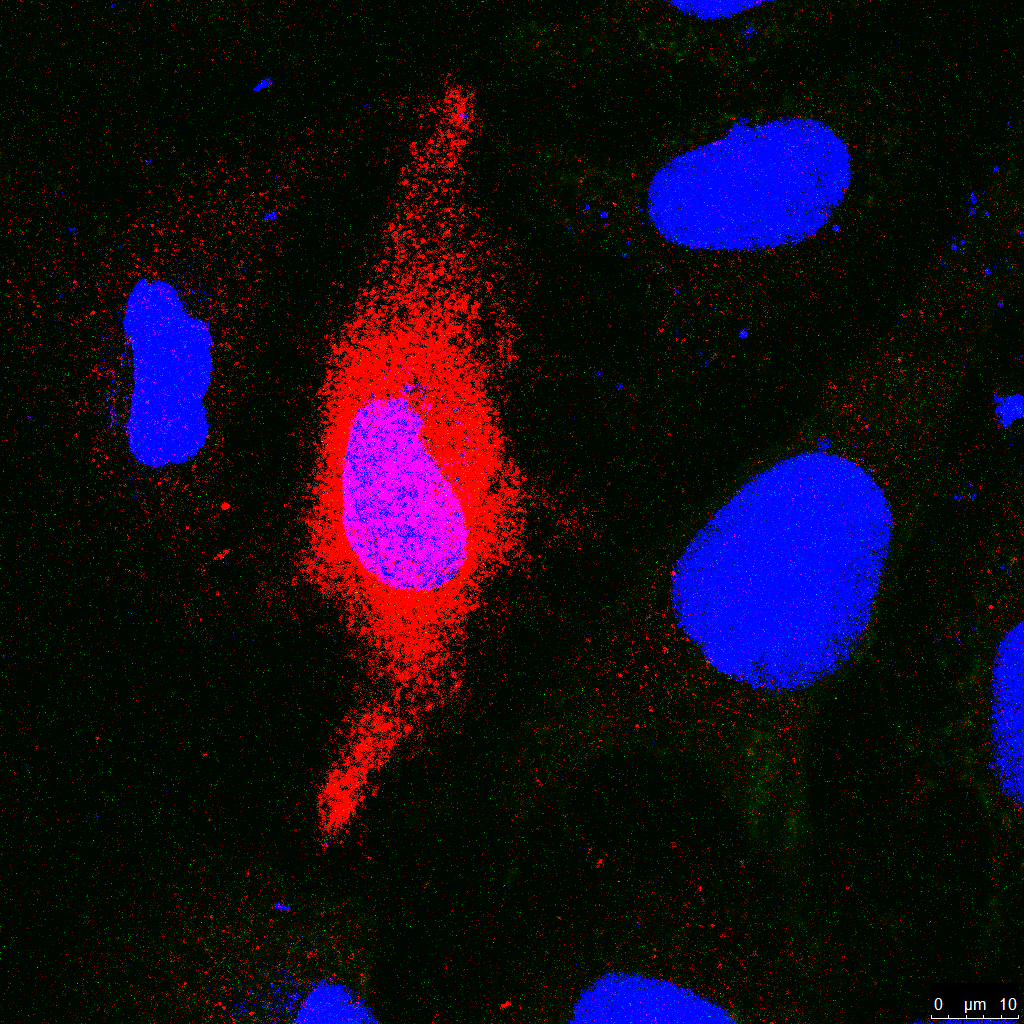

Supplement: Supplementary file 1 [file life-15-00714-s001.zip › Fig.3/fig.3-C/Experiment_SelM-12-2_z0.tif]

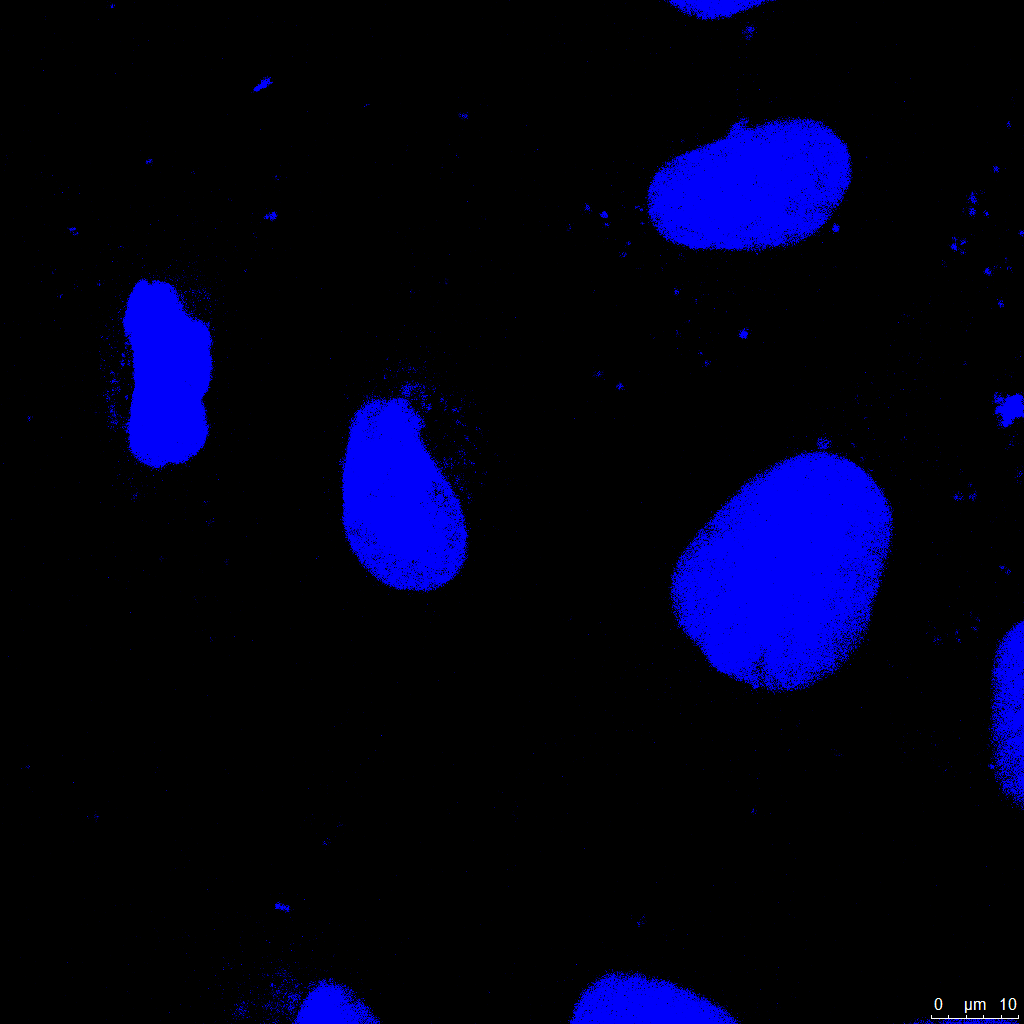

Supplement: Supplementary file 1 [file life-15-00714-s001.zip › Fig.3/fig.3-C/Experiment_SelM-12-2_z0_ch00.tif]

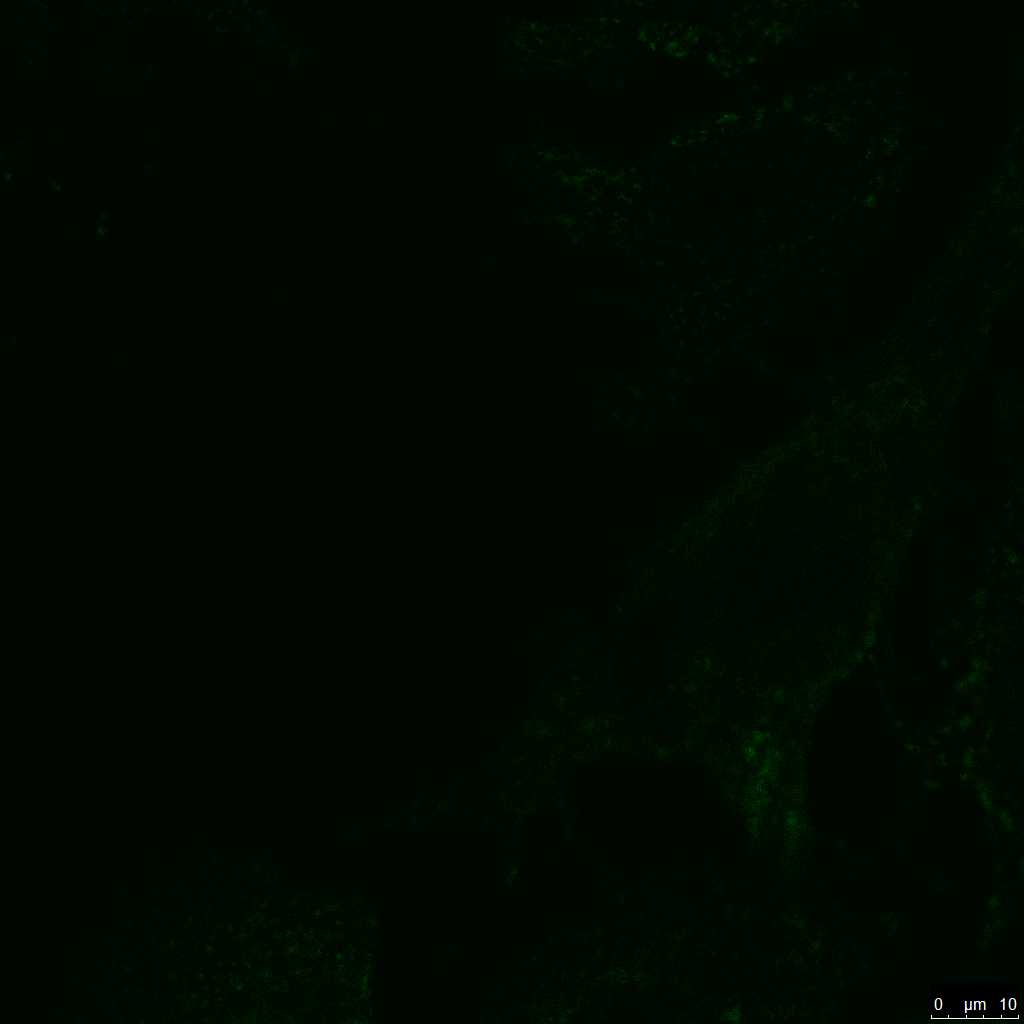

Supplement: Supplementary file 1 [file life-15-00714-s001.zip › Fig.3/fig.3-C/Experiment_SelM-12-2_z0_ch01.tif]

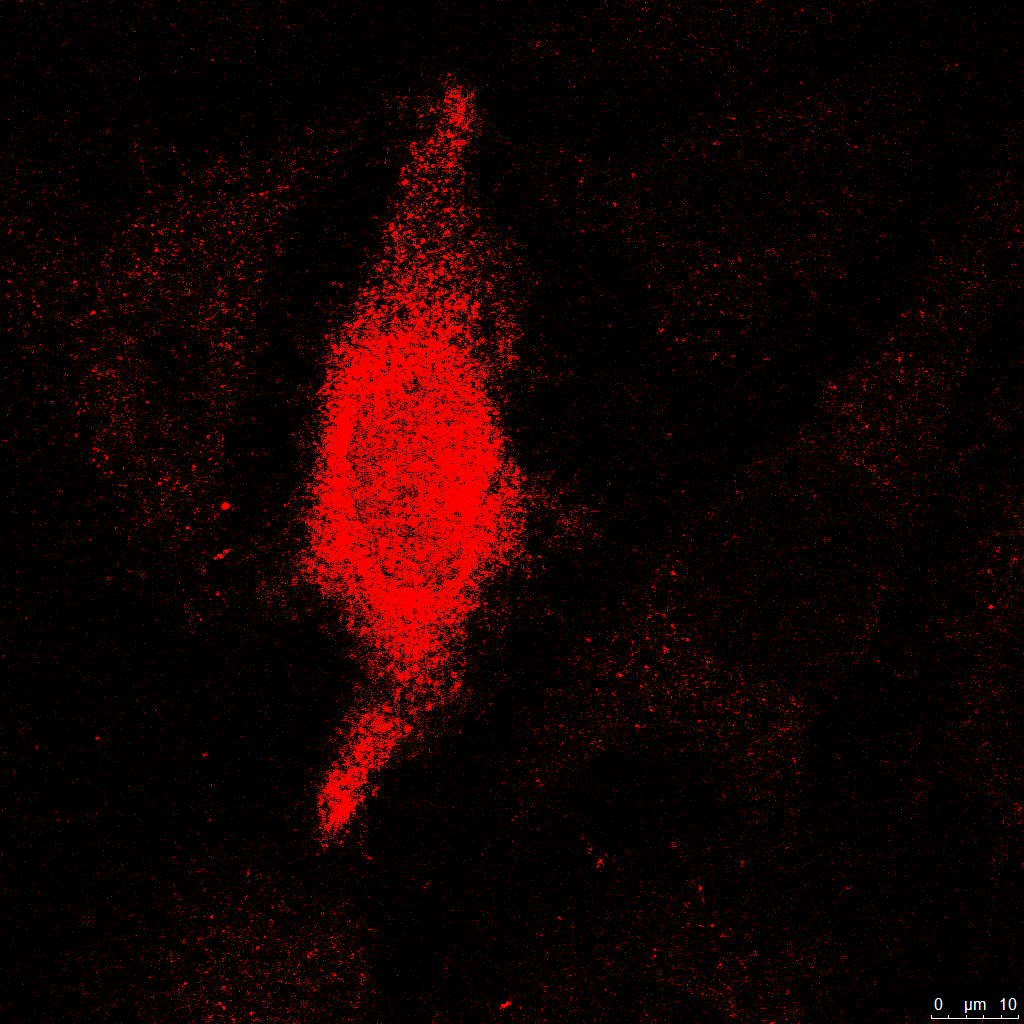

Supplement: Supplementary file 1 [file life-15-00714-s001.zip › Fig.3/fig.3-C/Experiment_SelM-12-2_z0_ch02.tif]

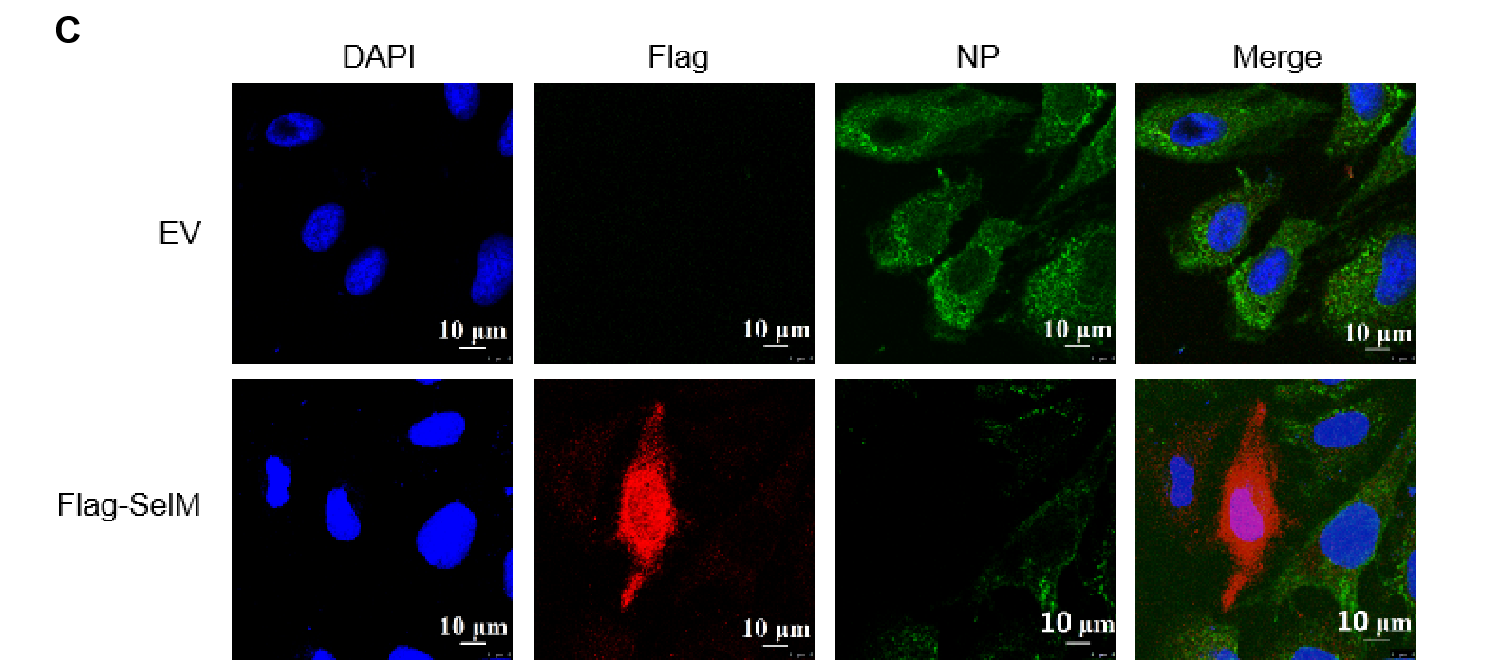

Supplement: Supplementary file 1 [file life-15-00714-s001.zip › Fig.3/fig.3-C/fig.3-C.tif]

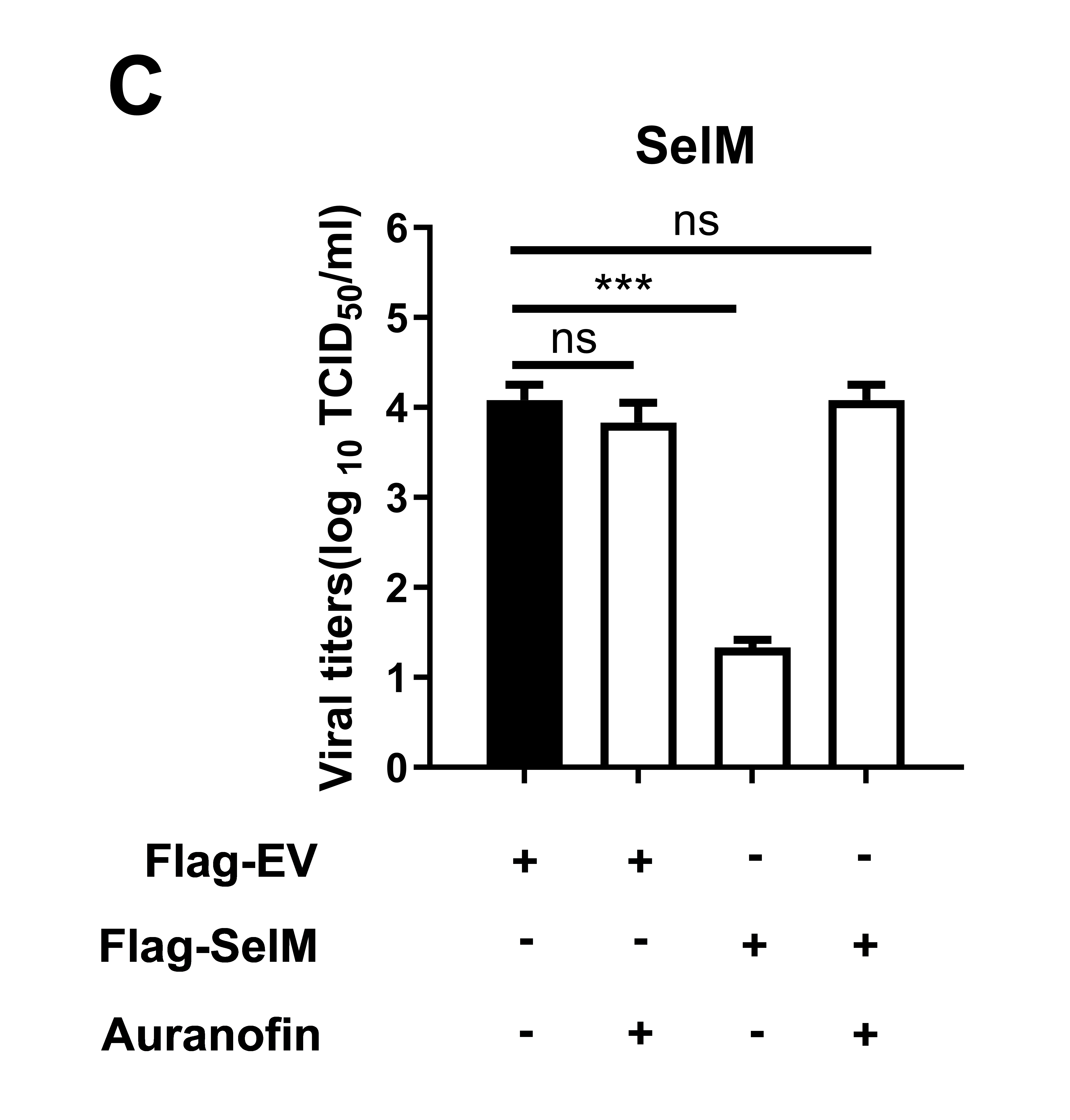

Supplement: Supplementary file 1 [file life-15-00714-s001.zip › Fig.4/Fig,4-C.tif]

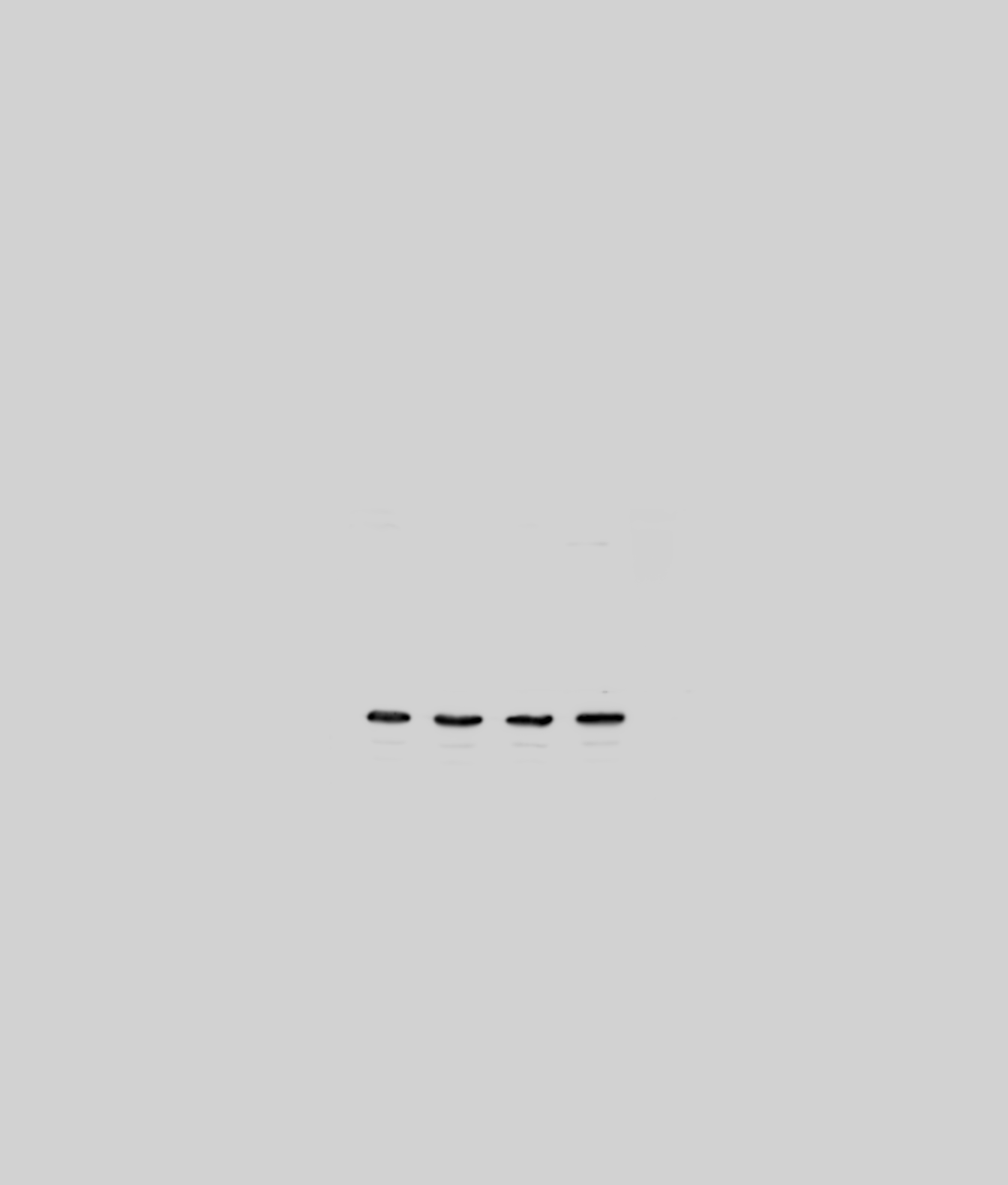

Supplement: Supplementary file 1 [file life-15-00714-s001.zip › Fig.5/Fig.5-A(2)/GAPDH-2-sample.tif]

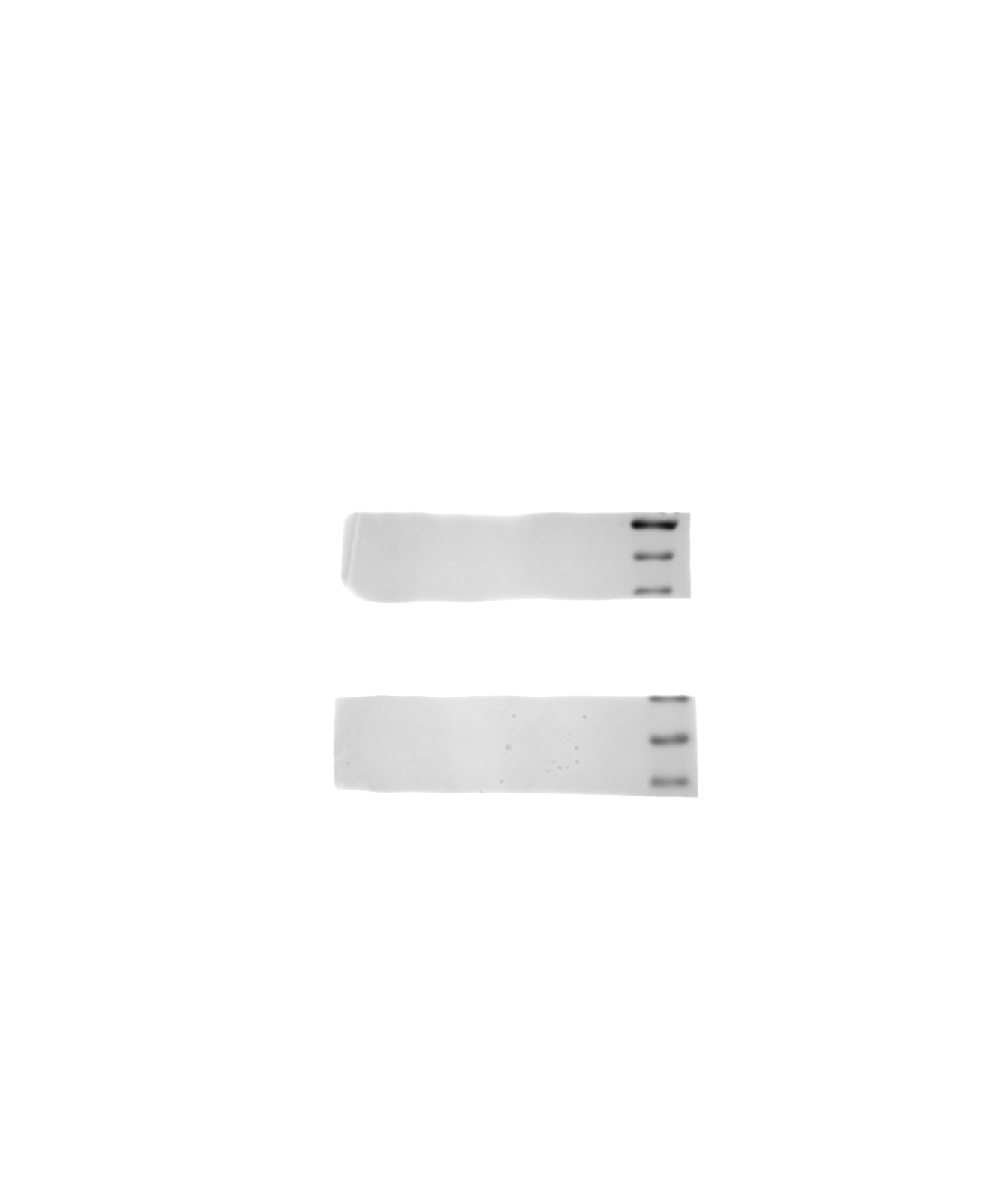

Supplement: Supplementary file 1 [file life-15-00714-s001.zip › Fig.5/Fig.5-A(2)/MAKER-marker.tif]

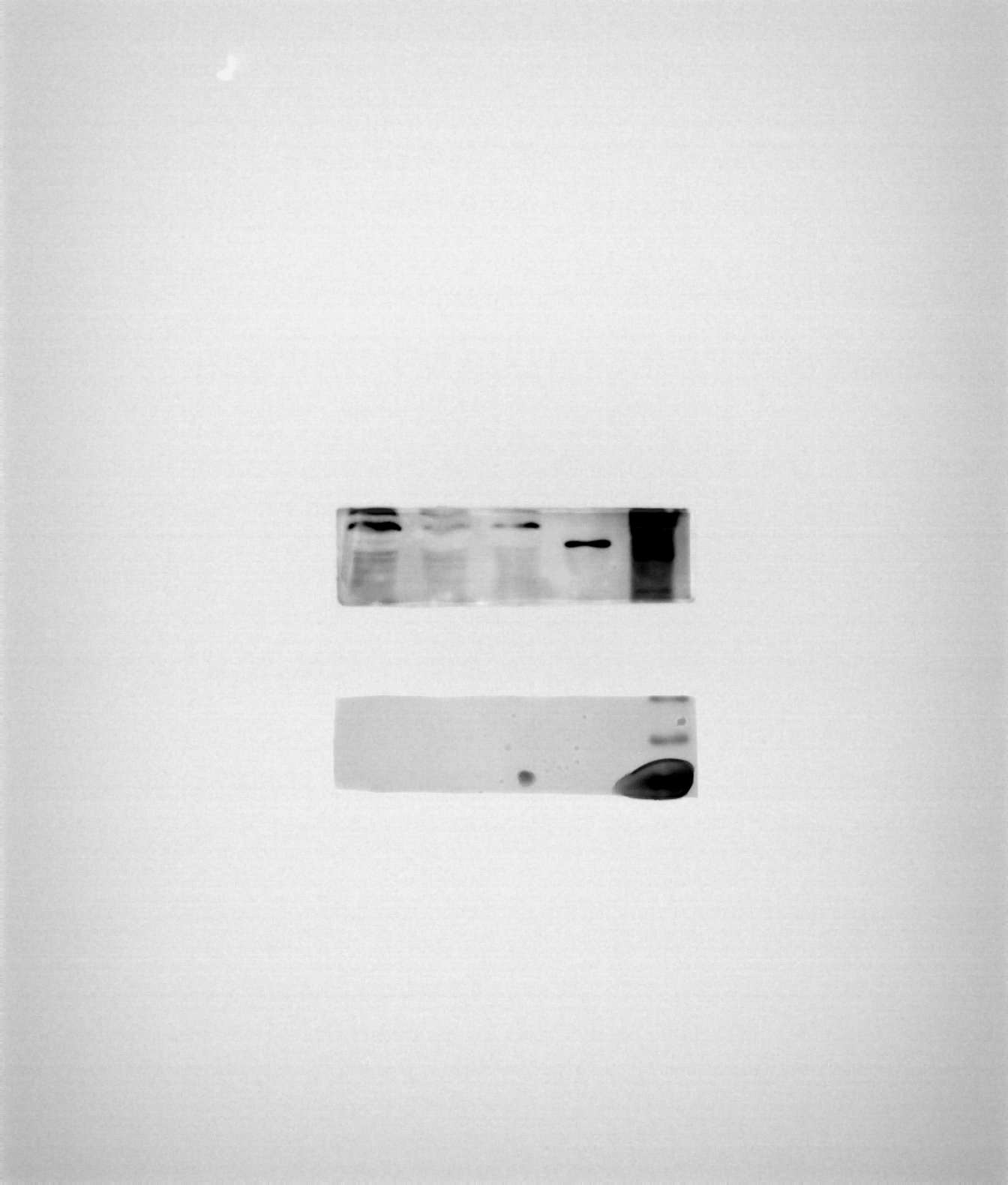

Supplement: Supplementary file 1 [file life-15-00714-s001.zip › Fig.5/Fig.5-A(2)/SelM mut-20S.tif]

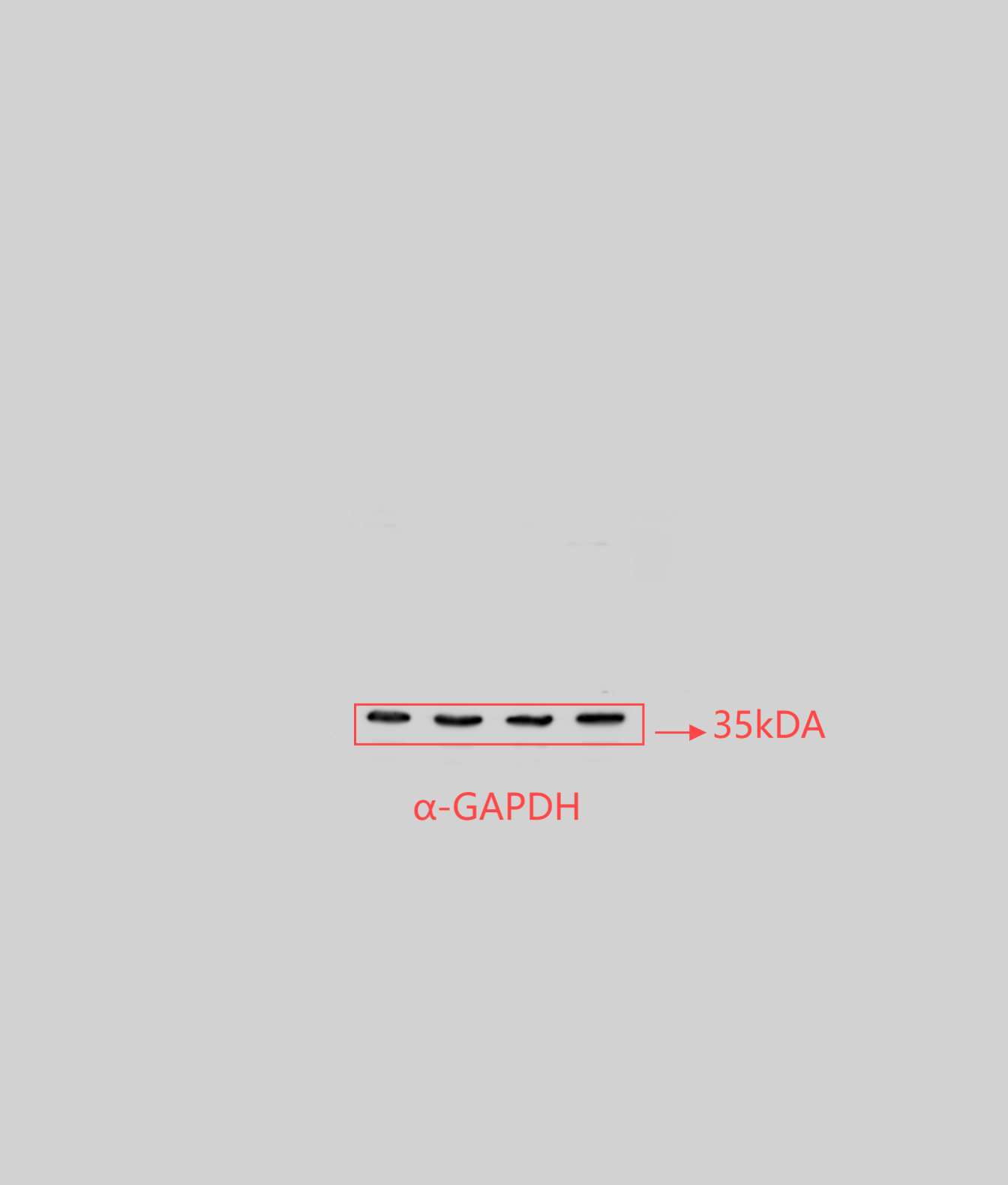

Supplement: Supplementary file 1 [file life-15-00714-s001.zip › Fig.5/Fig.5-A(2)/微信图片_20250423115328.png]

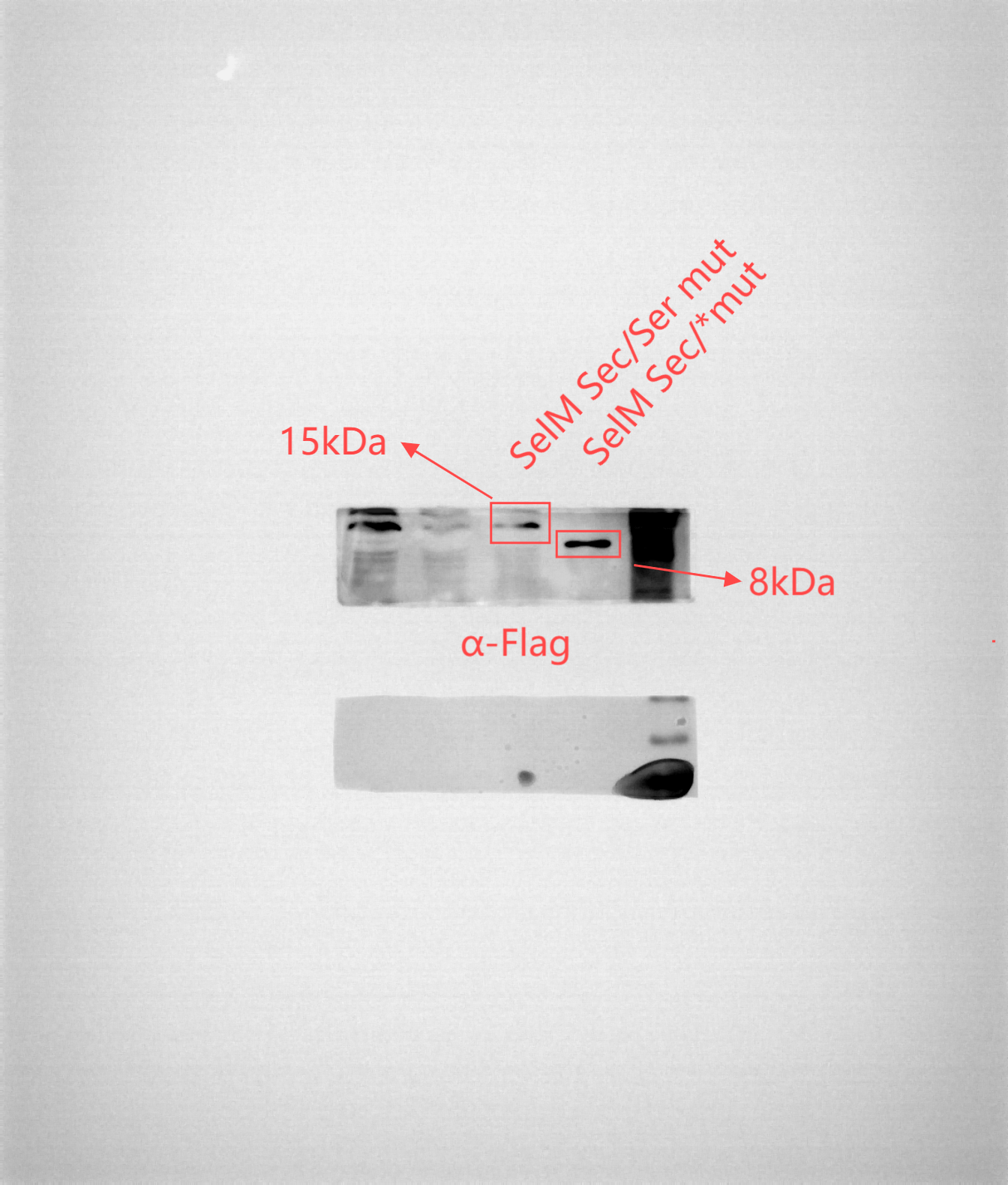

Supplement: Supplementary file 1 [file life-15-00714-s001.zip › Fig.5/Fig.5-A(2)/微信图片_20250423115340.png]

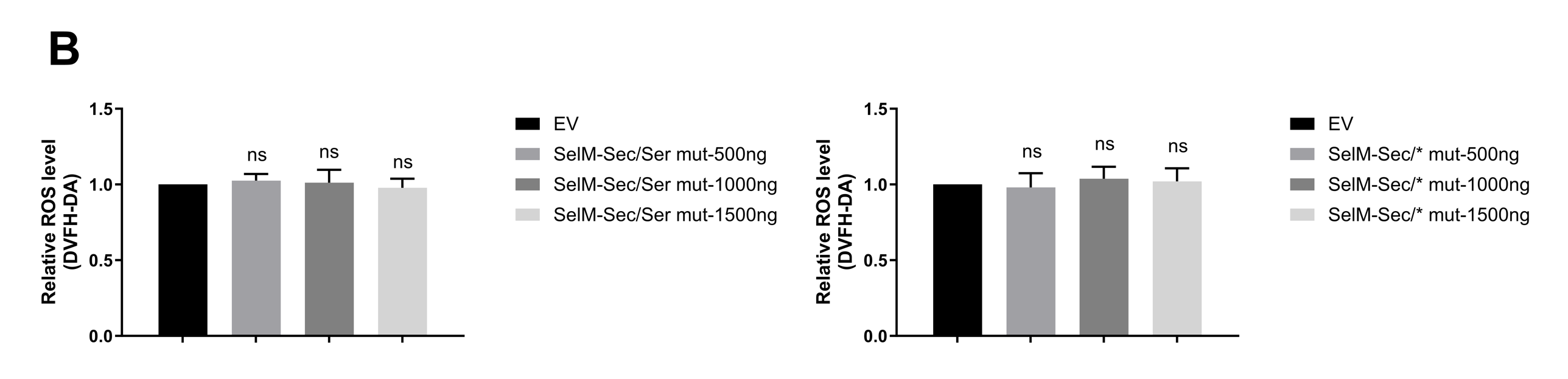

Supplement: Supplementary file 1 [file life-15-00714-s001.zip › Fig.5/Fig.5-B.tif]

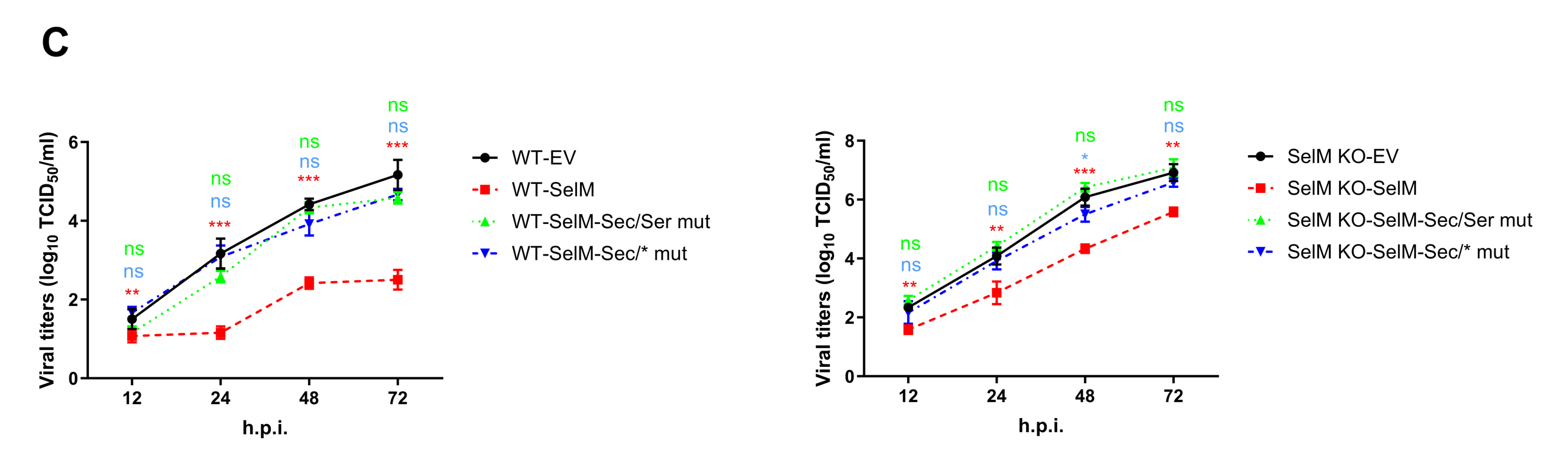

Supplement: Supplementary file 1 [file life-15-00714-s001.zip › Fig.5/Fig.5-C.tif]

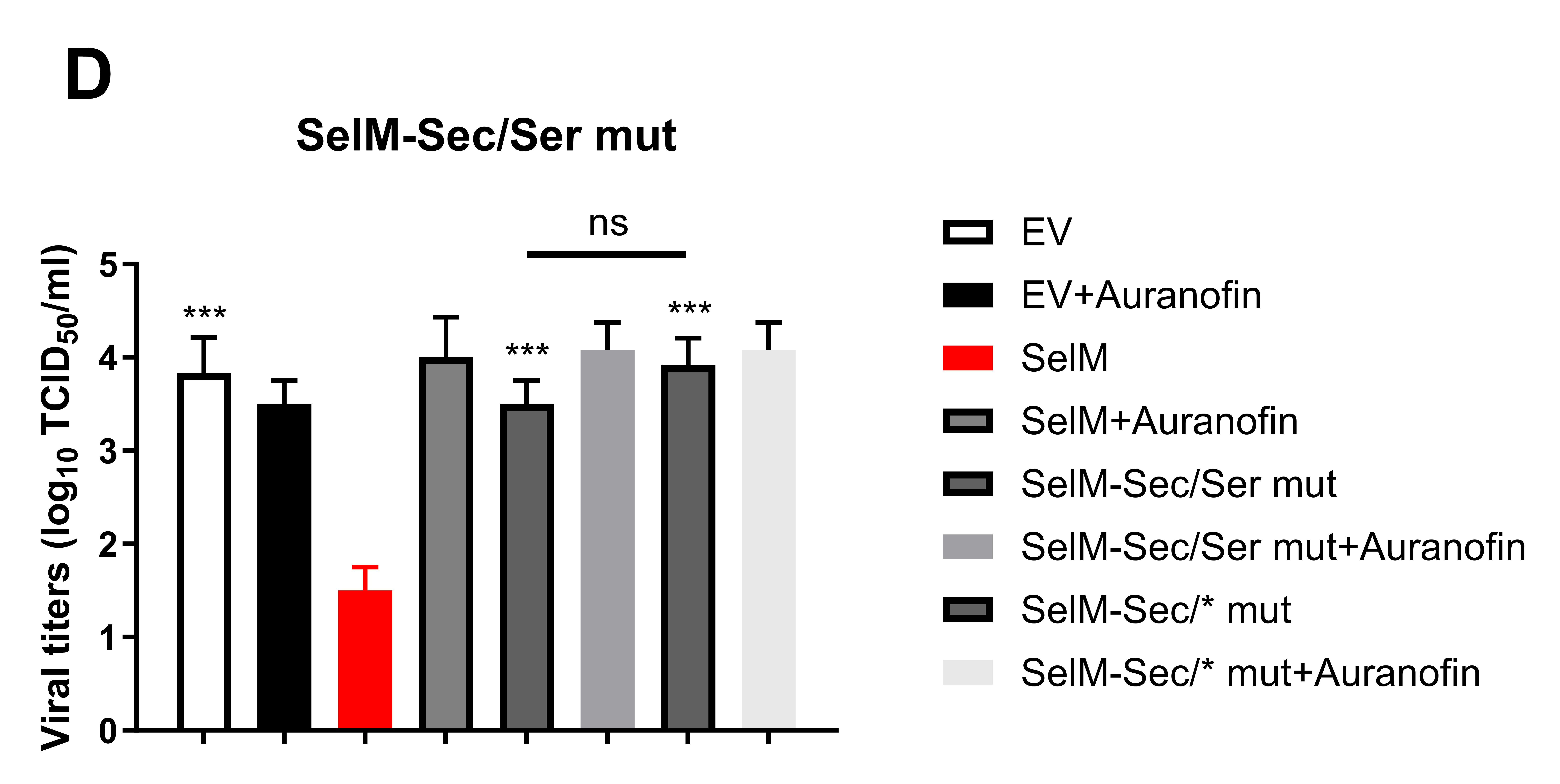

Supplement: Supplementary file 1 [file life-15-00714-s001.zip › Fig.5/Fig.5-D.tif]
